# Genome-wide regulation of KSHV RNA splicing by viral RNA-binding protein ORF57

**DOI:** 10.1371/journal.ppat.1010311

**Published:** 2022-07-14

**Authors:** Vladimir Majerciak, Beatriz Alvarado-Hernandez, Alexei Lobanov, Maggie Cam, Zhi-Ming Zheng

**Affiliations:** 1 Tumor Virus RNA Biology Section, HIV Dynamics and Replication Program, Center for Cancer Research (CCR), National Cancer Institute, NIH, Frederick, Maryland, Unites States of America; 2 CCR Collaborative Bioinformatics Resource, National Cancer Institute, NIH, Bethesda, Maryland, Unites States of America; Florida State University, UNITED STATES

## Abstract

RNA splicing plays an essential role in the expression of eukaryotic genes. We previously showed that KSHV ORF57 is a viral splicing factor promoting viral lytic gene expression. In this report, we compared the splicing profile of viral RNAs in BCBL-1 cells carrying a wild-type (WT) versus the cells containing an ORF57 knock-out (57KO) KSHV genome during viral lytic infection. Our analyses of viral RNA splice junctions from RNA-seq identified 269 RNA splicing events in the WT and 255 in the 57KO genome, including the splicing events spanning large parts of the viral genome and the production of vIRF4 circRNAs. No circRNA was detectable from the PAN region. We found that the 57KO alters the RNA splicing efficiency of targeted viral RNAs. Two most susceptible RNAs to ORF57 splicing regulation are the K15 RNA with eight exons and seven introns and the bicistronic RNA encoding both viral thymidylate synthase (ORF70) and membrane-associated E3-ubiquitin ligase (K3). ORF57 inhibits splicing of both K15 introns 1 and 2. ORF70/K3 RNA bears two introns, of which the first intron is within the ORF70 coding region as an alternative intron and the second intron in the intergenic region between the ORF70 and K3 as a constitutive intron. In the WT cells expressing ORF57, most ORF70/K3 transcripts retain the first intron to maintain an intact ORF70 coding region. In contrast, in the 57KO cells, the first intron is substantially spliced out. Using a minigene comprising of ORF70/K3 locus, we further confirmed ORF57 regulation of ORF70/K3 RNA splicing, independently of other viral factors. By monitoring protein expression, we showed that ORF57-mediated retention of the first intron leads to the expression of full-length ORF70 protein. The absence of ORF57 promotes the first intron splicing and expression of K3 protein. Altogether, we conclude that ORF57 regulates alternative splicing of ORF70/K3 bicistronic RNA to control K3-mediated immune evasion and ORF70 participation of viral DNA replication in viral lytic infection.

## Author summary

Like other herpesviruses, the expression of KSHV genes is in a tightly regulated manner during viral infection. The contribution of RNA splicing in the regulation of KSHV gene expression was extensively investigated in this report. We found that approximately one-third of all viral genes express spliced transcripts of which many undergo RNA alternative splicing. We identified a subset of viral RNA splicing events susceptible to the regulation of viral RNA-binding protein ORF57. This led to the discovery of ORF57-mediated regulation of the bicistronic ORF70/K3 RNA splicing and production of viral thymidylate synthase ORF70 and viral E3-ubiquitin ligase K3. Such regulation allows timely but mutually exclusive expression of these two viral proteins to execute their unique roles in immune evasion and viral genome replication during the KSHV lytic cycle. Thus, these data provide new insights into KSHV biology. Finally, our study represents a blueprint for RNA splicing studies of other pathogens in infected cells.

## Introduction

Kaposi’s sarcoma-associated herpesvirus (KSHV, or human herpesvirus 8, HHV-8) is considered as an etiological cause of several malignancies, including Kaposi’s sarcoma, primary effusion lymphoma (PEL), and multicentric Castleman’s disease [1–3]. KSHV DNA genome encodes up to a hundred viral genes being expressed in two functionally distinct transcriptional modes: latent and lytic, each defined by a specific subset of genes with distinct functions in the viral life cycle [4]. The KSHV transcriptome was the subject of numerous studies ranging from single gene locus mapping to genome-wide studies [5–11]. Collectively, these studies form the basis for our current understanding of viral gene expression and its contribution to KSHV pathogenesis. However, many aspects of viral gene expression regulation by viral and host factors remain to be understood.

RNA splicing plays an essential role in eukaryotic gene expression and almost all human pre-mRNAs are spliced. RNA splicing has been observed in transcripts of numerous viruses, including herpesviruses [12,13]. However, the number of split genes varies among individual herpesvirus family members, from only a few in α-herpesviruses to an estimated ~30% in KSHV [9,14]. The level of RNA splicing complexity varies among individual KSHV transcripts, with some exhibiting a splicing pattern resembling cellular constitutive RNA splicing while others show a highly complex alternative RNA splicing. Functionally, RNA splicing occurs in both coding and untranslated regions (UTR) of KSHV transcripts and regulates viral RNA export, stability of different isoforms of RNAs, and production of proteins and alternative ORFs [15,16].

Splicing of viral RNAs is mediated by host splicing machinery and thus subjects to regulation by host factors, including snRNA components of the cellular spliceosome and host splicing factors. The contribution of a viral factor(s) to RNA splicing was a long time suspected but not systematically studied. We previously showed that KSHV ORF57, a viral RNA-binding protein, acts as a viral splicing factor to promote the splicing of several KSHV immediate-early and early transcripts, including ORF50 and K8 [17]. To promote viral splicing, ORF57 interacts with components of host splicing machinery [17]. For example, we showed that ORF57 binds to cellular SRSF3 to relieve its suppressive activity on splicing of K8 suboptimal intron 2 [18], thus preventing the expression of the dominant-negative truncated K8β protein [19]. However, how ORF57 regulates comprehensive RNA splicing in KSHV genome expression remains to be investigated.

The introduction of next-generation sequencing has provided an opportunity to study RNA splicing events across the entire transcriptome. This study aims to provide an unbiased splicing-focused analysis of KSHV transcripts based on RNA-seq analysis of primary effusion lymphoma (PEL) BCBL-1 cells, a patient-derived KSHV-transformed B-cell line [20]. The mapped viral RNA splicing events were analyzed in the context of annotated KSHV genes. To determine the role of ORF57 in KSHV RNA splicing, we compared the splicing of viral RNAs in BCBL-1 cells containing a WT KSHV genome with the BCBL-1 cells containing an ORF57-null KSHV genome generated by a modified CRISPR/Cas9 knock-out technology [21]. This analysis led to identify KSHV splicing events susceptible to ORF57-dependent regulation, including alternative splicing of ORF70/K3 bicistronic transcripts and to uncover a new mode regulation of KSHV gene expression by viral ORF57.

## Results

### Viral gene expression in BCBL-1 single-cell clones with or without KSHV ORF57 expression

**Fig 1:**
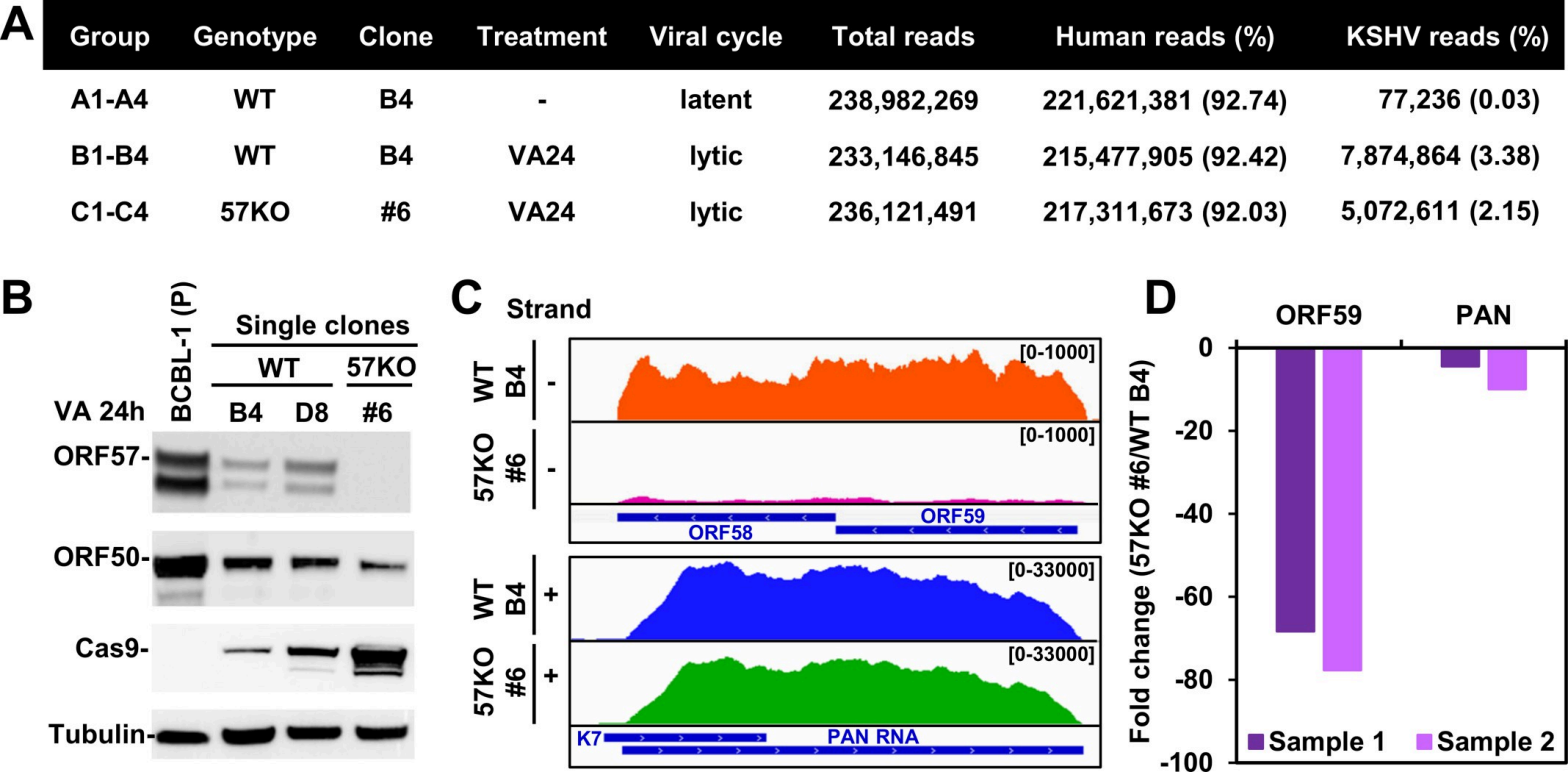
Viral gene expression in BCBL-1 single-cell clones. (A) Samples used for RNA-seq including WT single-cell clone B4 of BCBL-1 cells carrying Cas9 vector lacking gRNAs expression and 57KO single-cell clone #6 carrying a plasmid expressing gRNA2+4 (pABC8) described in previous studies [21]. Total RNA from uninduced clone B4 cells representing WT cells with latent infection (4 samples, A1-4), clone B4 cells treated with 1 mM valproic acid for 24 h (VA24) representing the WT cells with the lytic infection (4 samples, B1-4), and clone #6 cells treated with 1 mM valproic acid for 24 h (VA24) representing the 57KO cells with the lytic infection (4 samples, C1-4) were subjected to RNA-seq. Total RNA-seq reads uniquely mapped to the human (hg38)/KSHV (GenBank Acc. No. HQ404500) chimeric genome were counted in each group of four samples. (B) Relative protein expression of ORF57, ORF50, and Cas9 in parental BCBL-1 (P) and BCBL-1 single-cell clones containing a wild-type genome (WT clones B4 and D8) or an ORF57-null genome (57KO clone #6) under 24 h of KSHV lytic infection induced by 1 mM valproic acid (VA 24h). Immunoblot was performed with each protein corresponding specific antibody, with β-tubulin serving as a protein loading control. (C) RNA-seq reads-coverage of KSHV ORF58-59 and PAN RNA loci in one representative sample each from WT B4 and 57KO #6 cells with KSHV lytic infection induced by 24 h VA treatment. The numbers in the upper right corner represent the reads-coverage depth. (D) Validation of PAN and ORF59 RNA expression from WT B4 and 57KO #6 cells by RT-qPCR. Total RNA from WT B4 and 57KO #6 cells with KSHV lytic infection described in panel C was used for the assays.

To perform a comprehensive genome-wide analysis of KSHV RNA splicing and its regulation by ORF57, we carried out RNA-seq of total RNA isolated from BCBL-1 single-cell clones carrying a wild-type (WT, clone B4) or ORF57 knock-out (57KO, clone #6) KSHV genome 24 h after induction of viral lytic replication with valproic acid (VA24) [21] (**Fig 1A**). Interestingly, for an unknown reason, all selected single-cell WT clones transfected with the empty Cas9 vector express a much lower amount of ORF57 protein when compared to the parental BCBL-1 cells but still support KSHV lytic replication (**Fig 1B)**. We selected the WT B4 clone, instead of the WT B8 cells or even the parental BCBL-1 cells, for parallel RNA-seq comparison with the 57KO clone #6 cells because the WT B4 clone cells expressed the lowest amount of ORF57 essential for KSHV lytic replication [21,22] and thus could give us a similar amount of viral RNA reads for unambiguous RNA splicing analysis of viral RNA transcripts most sensitive to ORF57 regulation. Principal component analysis of differentially expressed genes from three group samples in RNA-seq showed well-separated expression profiles for each group, indicating the high quality of the cDNA library preparation and RNA-sequencing. Each group of four samples exhibited 239 (WT B4 latent), 233 (WT B4 lytic), and 236 (57KO #6 lytic) million combined high-quality RNA-seq reads (**Fig 1A**). By mapping the sequence libraries to a host GRCh38 (hg38)/BCBL-1 KSHV (GenBank Acc. No. HQ404500) chimeric genome using STAR aligner [23], we found the reduced expression of ORF58 and ORF59 in the 57KO cells when compared with WT B4 cells, as expected [24,25] (**Fig 1C and S1 Table**). Surprisingly, there was only a slight expression decrease (1.3-fold, p <0.01) for PAN RNA from the 57KO #6 cells to WT B4 cells (**Fig 1C and S1 Table**), despite that PAN RNA has been identified as a sensitive downstream target of ORF57 [24,26]. We subsequently verified the RNA-seq results by quantitative RT-qPCR (**Fig 1D**). We showed a ~73 (68–78)-fold reduction of ORF59 and only ~7 (4–10)-fold reduction of abundant PAN RNA in the 57KO #6 cells when compared with the WT B4 cells. Data suggest an unknown compensatory mechanism for PAN RNA expression in the absence of ORF57 in the course of long-term selection of single-cell clones.

### Mapping of KSHV RNA splice junctions from the WT KSHV to ORF57-null KSHV genome in BCBL-1 cells

**Fig 2:**
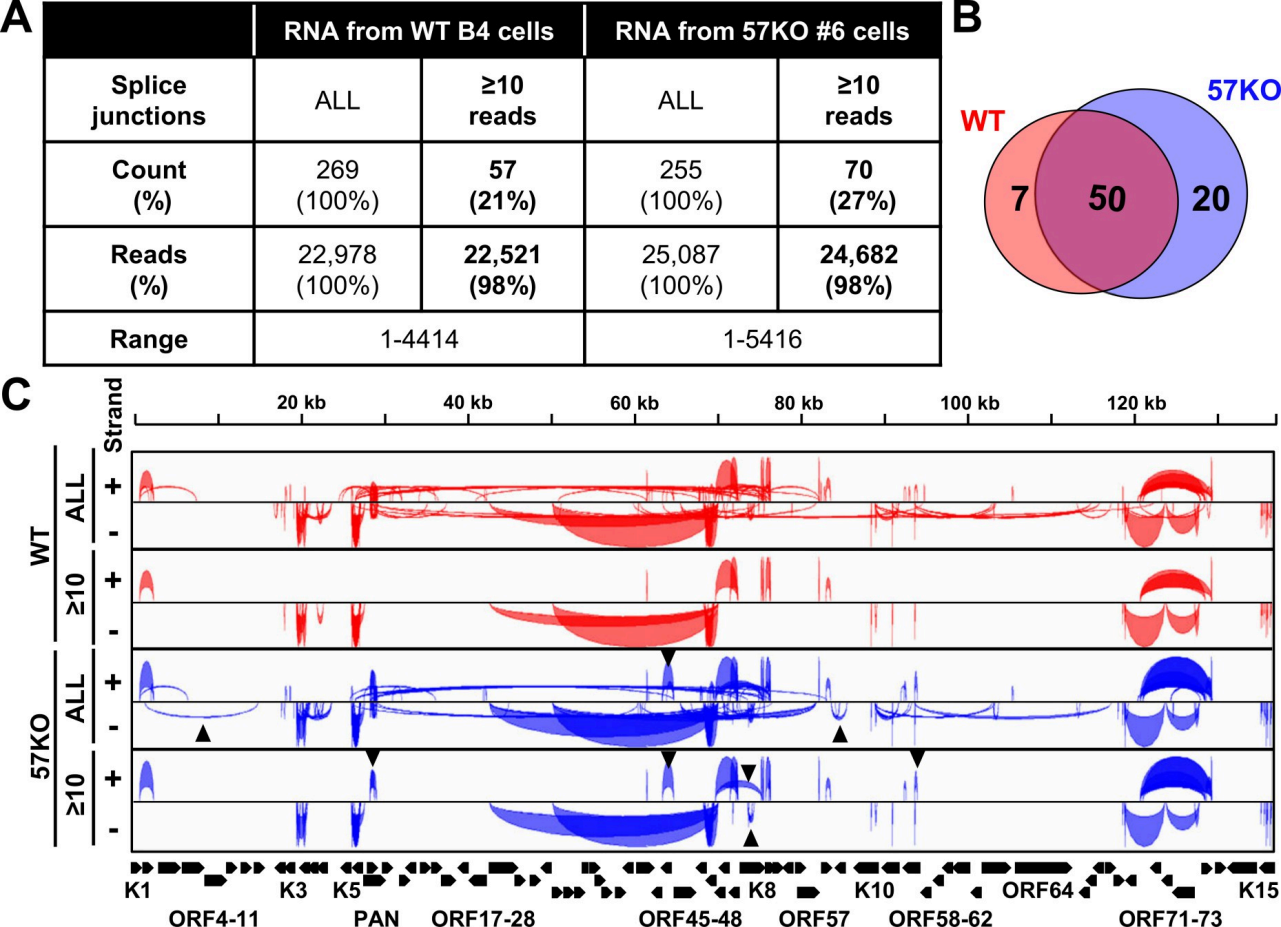
Identification of KSHV RNA splice junctions by RNA-seq. (A) The number (count) of all detected KSHV RNA splice junction and those with the splice reads (≥10 reads) identified by STAR aligner [23] in WT B4 and 57KO #6 cells (combined 4 samples in each group) with lytic KSHV infection induced by 24 h treatment with 1 mM valproic acid. “Reads” are the number and percentage (%) of all supporting splice reads from all splice junctions. (B) The Venn diagram shows the overlapped RNA splice junctions with ≥10 supporting splice reads between the WT and 57KO genome. (C) The Sashimi graphs generated by IGV showing the viral genomic locations and frequency of RNA splicing events in individual groups. The black triangles indicate the notable RNA splicing differences between the 57KO and WT genome in the cells.

As our previous study showed high reliability in qualitative and quantitative analysis of genome-wide RNA splicing of mouse papillomavirus (MmuPV-1) [27] by using STAR aligner [23], the splice reads uniquely mapped to the KSHV genome were extracted, pooled together, and used for viral RNA splicing analysis. Because both groups exhibited an almost equal sequence library size, we did not perform any further read counts normalization. In total, we identified 22,978 viral RNA splice junction reads derived from 269 viral splice junctions in the cells with the WT genome and 25,087 viral RNA splice junction reads derived from 255 viral splice junctions in the cells with the 57KO genome (**Fig 2A**). Among those viral RNA splice junctions, 145 were commonly detected in both WT and 57KO cells. However, many identified viral RNA splice junctions were supported with only a few reads and were not consistently detected in all samples within individual groups, indicating they represent rare, low-frequency RNA splicing events (see details in **S2 Table**). Therefore, we next focused only on more prevalent viral RNA splice junctions supported by at least 10 or more (≥10) splice reads. Even though applying this threshold led to ~5-fold reduction of the number of viral RNA splice junctions to 57 in the cells with the WT genome and 70 in the cell with the 57KO genome, the splice reads assigned to these junctions counted for 98% of all identified viral splice reads, indicating that these RNA splice junctions represent most of the viral RNA splicing events (**Fig 2A**) with 54 in the WT and 64 in the 57KO consistently being detected in all four samples in each group (**S2 Table**). As expected, the highly prevalent splice junctions were found in viral RNAs from the WT to 57KO genome, with 50 viral RNA splice junctions commonly detected in the viral RNAs expressed from both genomes. The remaining 7 viral RNA splice junctions passed the minimal 10 reads threshold only in the WT and the other 20 only in the 57KO genome (**Fig 2B**).

Mapping the obtained splice junctions to the reference KSHV genome revealed a landscape of viral RNA splicing with clusters of split genes being separated by the genomic regions lacking splice sites (**Fig 2C**). We also observed several noticeable differences between RNA splicing from the WT to the 57KO genome (**Fig 2C**). To determine the biological relevance of the detected viral splicing events, all highly prevalent splice junctions were mapped to each annotated KSHV transcripts with previously mapped or predicted transcriptional start sites (TSS) and polyadenylation cleavage sites [8–10]. The RNA-seq reads-coverage was used to estimate RNA splicing efficiency. Finally, the coding potential of individual spliced RNA isoforms was analyzed to predict the effect of RNA splicing on the viral proteome.

### KSHV RNA splicing profile from the WT genome in BCBL-1 cells

**Fig 3:**
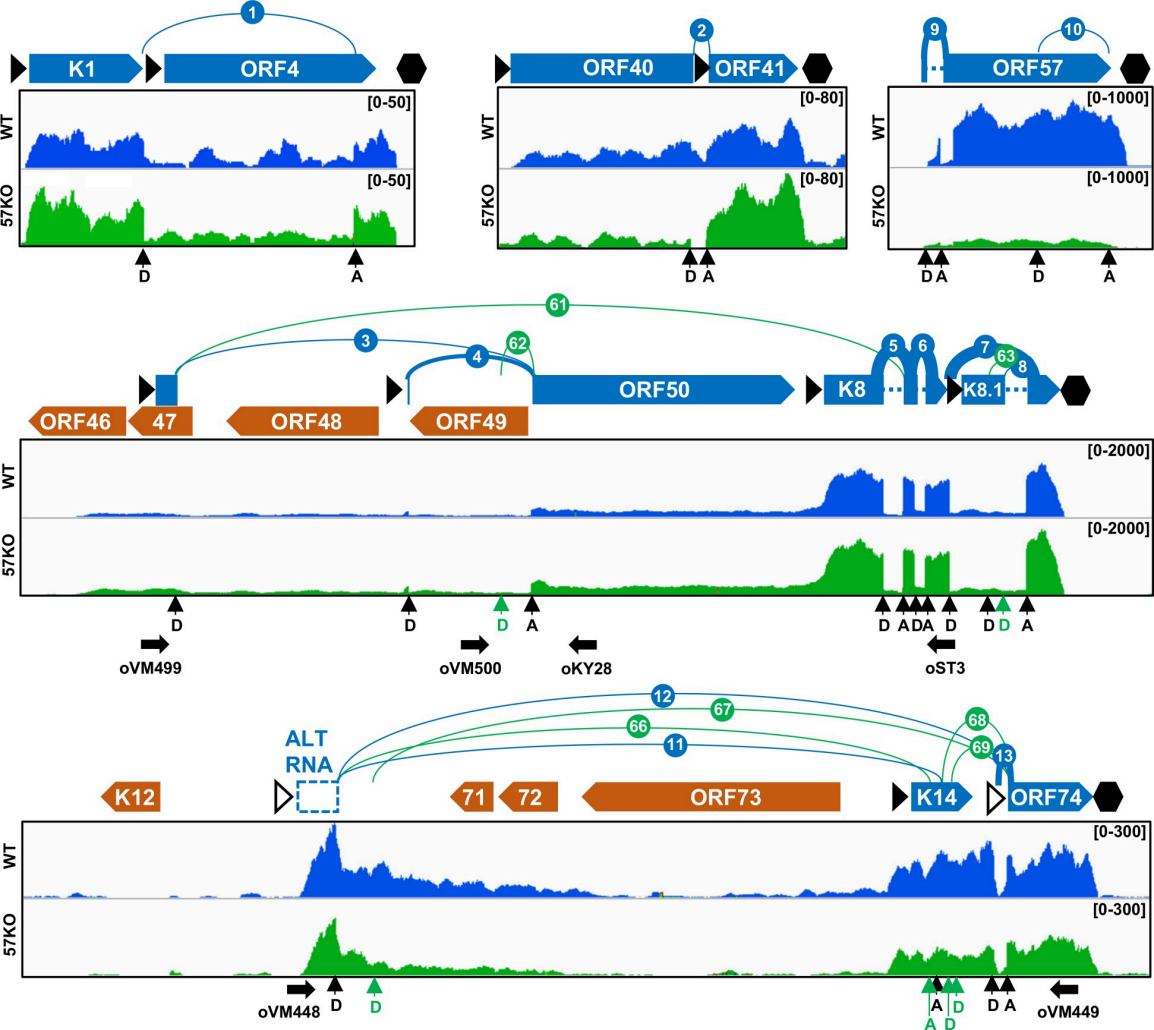
Mapping of KSHV RNA splice junctions from the plus strand of viral genome. The diagrams depicting all splice junctions (colored arches) with ≥10 supporting splice reads mapped to viral transcripts expressed from the plus strand of the KSHV genome, with the splicing events being numbered in the order from the genome 5′ to 3′ direction. The numbered blue arches represent splice junctions detected from the WT genome and green from the 57KO genome in the cells (see **Tables 1 and 3**). The arch thickness represents a relative abundance of detected splice junction reads. The RNA-seq reads-coverage from one representative sample from the WT and 57KO genome in the cells with lytic infection is shown below the arches, with the reads-coverage depth shown in the upper right corner. The annotated KSHV ORFs are marked by full arrows, blue for ORFs encoded from the plus or orange for ORFs from the minus DNA strand. The dashed boxes are predicted non-coding exons. The black triangles represent mapped (full) or predicted (empty) transcriptional start sites [9, 10]. The black hexagons mark the mapped polyadenylation cleavage sites [8]. The 5′ splice donor (D) and 3′ splice acceptor (A) sites are marked with vertical arrows. Horizontal arrows mark the positions of primer sets used in RT-PCR.

**Fig 4:**
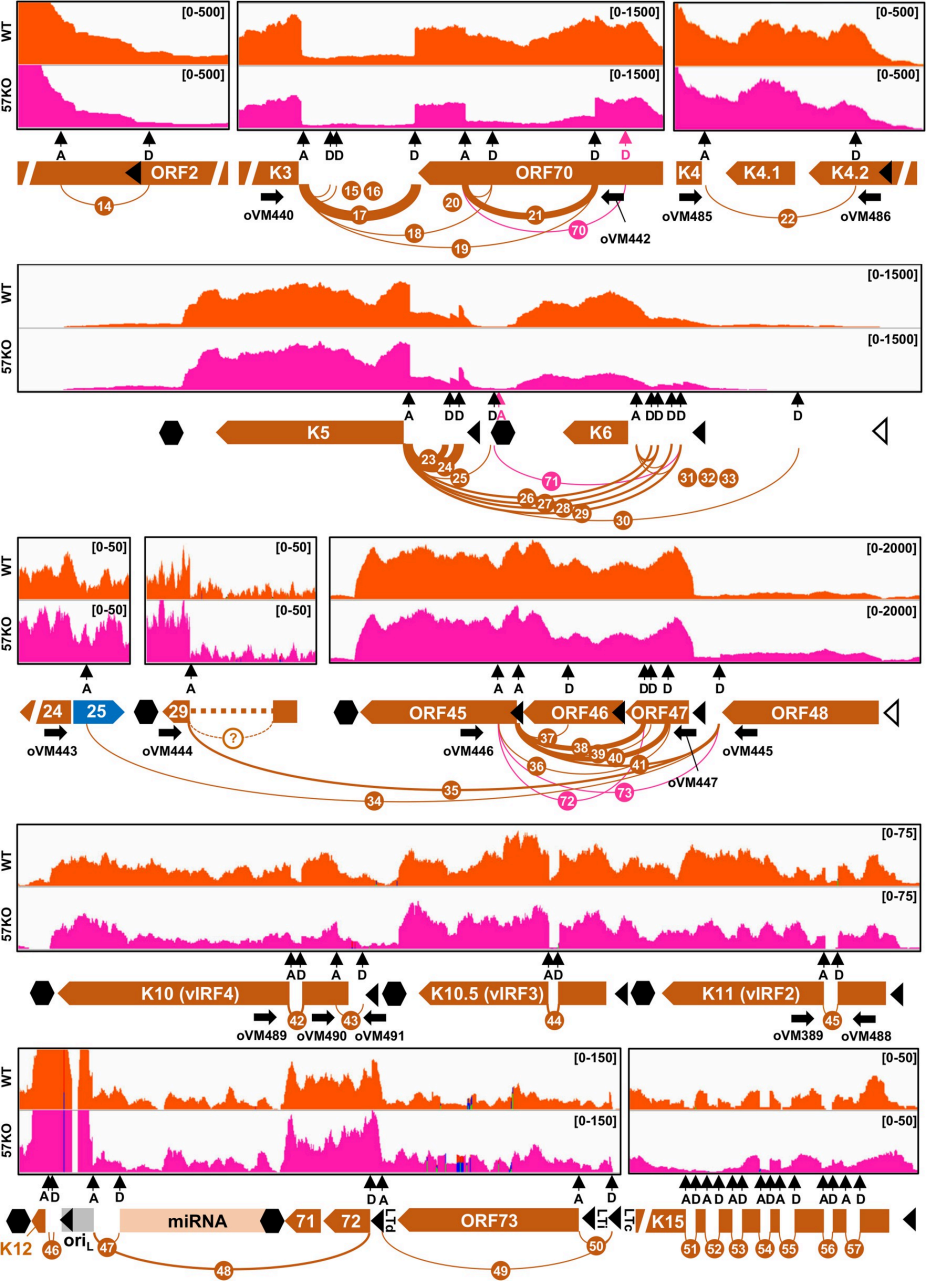
Mapping of KSHV RNA splice junctions from the minus strand of viral genome. The diagrams depicting all splice junctions (colored arches) with ≥10 supporting splice reads mapped to viral transcripts expressed from the minus strand of the KSHV genome, with the splicing events being numbered in the order from the genome 5′ to 3′ direction. The numbered orange arches represent splice junctions detected from the WT genome and pink arches from the 57KO genome in the cells (see **Tables 1 and 3**). The arch thickness represents a relative abundance of detected splice junction reads. The RNA-seq reads-coverage from one representative sample from the WT and 57KO genome in the cells with lytic infection is shown above the arches, with the reads-coverage depth shown in the upper right corner. See other details in **Fig 3**. LTc—constitutive latent promoter, LTi—inducible latent promoter, LTd—distal latent promoter, oriL—lytic origin of replication.

**Fig 5:**
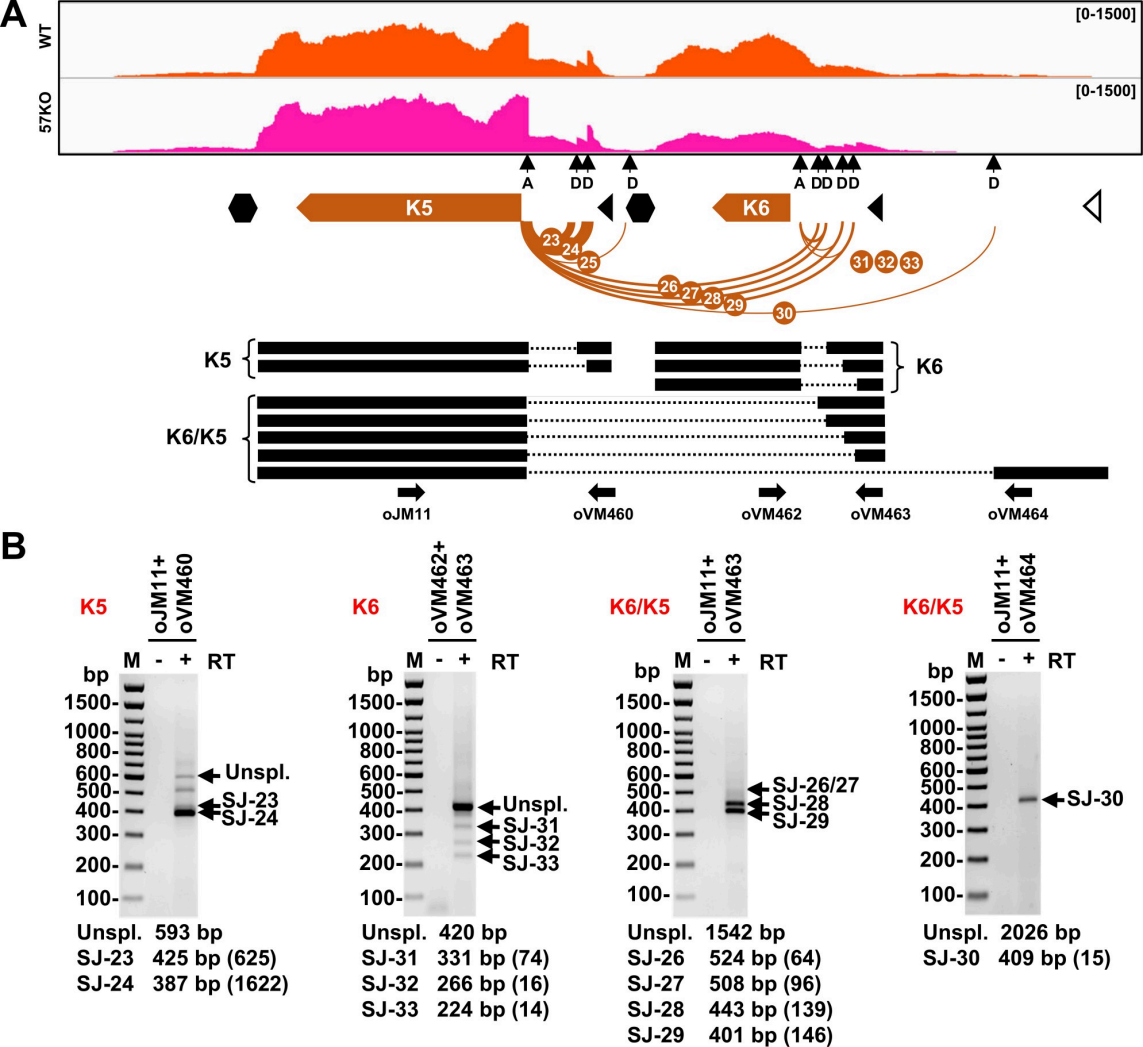
Alternative RNA splicing detected and verified from the K5/K6 locus. (A) The RNA splice junctions mapped to K5 and K6 locus in the WT genome. Below is the predicted structure of K5, K6, and K6/K5 transcripts with thick black lines representing the exons and dotted lines the introns. The arrows represent the primers used in RT-PCR shown in (B). See other details in **Fig 3**. (B) Verification of the mapped RNA splice junctions in K5/K6 expression in BCBL-1 cells with lytic KSHV infection. Total RNA extracted from the parental BCBL-1 cells with VA 24 h treatment was examined by RT-PCR. The reaction without reverse transcriptase (RT-) was used as a negative control. The predicted sizes of amplificons from unspliced and spliced transcripts are displayed under each gel electrograph. All spliced products amplified were gel purified and confirmed by sequencing. The number of supporting splice reads from the WT genome in the cells with lytic KSHV induction are in the parentheses.

All 57 highly prevalent splice junctions with ≥10 splice reads detected in viral RNAs from the WT genome were named SJ-1 to SJ-57 in the order from the KSHV genome 5′ to 3′ direction and are summarized in **Table 1**. They mapped to transcripts from 33 annotated KSHV genes (**Figs 3 and 4**), confirming the previous estimates that ~30% of KSHV genes are undergoing RNA splicing when expressed [14]. These include almost all previously reported splice junctions identified either in the single gene locus or in the gene cluster regions [9], but we were unable to detect any RNA splice junction within the ORF29 gene as reported [5]. In addition, several minor alternative RNA splice junctions previously reported in ORF4 and K8.1 RNAs were detected in this study but did not pass the threshold of ≥10 splice reads [28,29]. In contrast, we identified numerous novel splice junctions, some of which were previously detected in other RNA-seq studies but not properly annotated [30]. These include additional new splice junctions in viral transcripts of ALT RNA, ORF70/K3, K5, ORF45/48, K10, and a major latent locus, indicating a higher complexity of RNA splicing of these transcripts (**Figs 3 and 4**). We also identified several novel RNA splicing events in the genes that were not found to be spliced in previous studies. These include splice junctions in the ORF2 and K4.1/4.2 loci (**Fig 4**). Despite their relatively low prevalence, the RT-PCR results support the newly detected introns in the KSHV WT genome in BCBL-1 cells (**S1 Fig**). Other examples of novel splice junctions detected, validated, and functionally annotated in our study are the junctions between K5 encoding membrane-based E3-ubiquitin ligase and K6 encoding viral cytokine vMIP-I [31]. Interestingly, we identified that splice junctions could be not only mapped to individual K5 (SJ-23, -24, -25) and K6 (SJ-31, -32, -33) transcripts, but also five new splice junctions spanning over K6 gene to K5 (SJ-26, -27, -28, -29, -30) (**Fig 5A**). The existence of these splice junctions suggests the expression of not only monocistronic K6 and K5 transcripts but also K6/K5 bicistronic transcripts, which are further processed by RNA splicing. The alternative splicing in both K5 and K6 monocistronic RNAs occurs in their 5′ UTRs and is mediated by several alternative 5′ ss and single 3′ ss right upstream of their corresponding coding regions (**Fig 5A**). Interestingly, expression of several upstream ORFs (uORF) was detected in both 5′ UTRs [9]. Thus, the observed RNA splicing may regulate K5 and K6 expression by removing these uORFs to efficiently translate K5 and K6 proteins, which requires further experimental validations. The splicing of bicistronic RNA occurs between a 5′ ss upstream of the K6 ORF and a 3′ ss upstream of the K5 ORF. This RNA splicing removes the entire K6 code region without affecting the K5 coding capacity. Therefore, the spliced K6/K5 mRNA under control by the K6 promoter encodes only K5 protein (**Fig 5A**). The existence of these RNA splicing events was confirmed by RT-PCR (**Fig 5B**). Together these data reveal a comprehensive profile of KSHV genome-wide RNA splicing events during KSHV lytic infection.

**Table 1:** Identified RNA splice junctions (SJ) with ≥10 splice reads from the KSHV WT or 57KO genome in BCBL-1 cells. The positions of 5′ and 3′ splice sites (SS) are based on the KSHV reference genome GenBank Acc. No. HQ404500. The reads-number represents a total number of splice reads in the WT or 57KO group during lytic replication (see S2 Table for the details). The SJ fold change (FC) represents splice junction-reads change in BCBL-1 cells with the KSHV 57KO versus WT genome during viral lytic replication. The asterisk marks significant (p ≤ 0.05) change by Student *t*-test. The transcript FC for the annotated genes was determined by Limma Voom differential gene expression analysis (see S1 Table for the details), with the color gradient red for decrease and green for increase. The genes with significant expression changes (p ≤0.01) are marked with an asterisk. N/D—not determined.

Splice junction	5′ SS (nt position)	3′ SS (nt position)	Intron size (nt)	Strand	Reads		Transcript	SJ FC 57KO/WT	FC 57KO/WT
					WT	57KO			
**SJ-1**	981	2625	1643	+	28	42	**K1/ORF4**	1.50	1.23 (K1), -1.05 (ORF4)
**SJ-2**	61179	61307	127	+	28	27	**ORF40/41**	-1.04	-2.14*(ORF40), 1.14 (ORF41)
**SJ-3**	69364	72094	2729	+	46	72	**ORF50**	1.57	1.30 (ORF50)
**SJ-4**	71135	72094	958	+	467	649	**ORF50**	1.39	1.30 (ORF50)
**SJ-5**	74845	74993	147	+	4414	4860	**ORF50/K8**	1.10	1.30 (ORF50), 1.09 (K8)
**SJ-6**	75085	75167	81	+	3034	3796	**ORF50/K8**	1.25	1.30 (ORF50), 1.09 (K8)
**SJ-7**	75360	75955	594	+	3800	5416	**ORF50/K8**	1.43	1.30 (ORF50), 1.09 (K8)
**SJ-8**	75860	75955	94	+	49	42	**ORF50/K8/K8.1**	-1.16	1.30 (ORF50), 1.09 (K8), 1.03 (K8.1)
**SJ-9**	81640	81749	108	+	1885	172	**ORF56/ORF57**	-11.11*	-1.65 (ORF56), -9.02* (ORF57)
**SJ-10**	82497	83069	571	+	17	15	**ORF56/ORF57**	-1.14	-1.65 (ORF56), -9.02* (ORF57)
**SJ-11**	119871	127583	7711	+	29	25	**ALT RNA**	-1.16	N/D
**SJ-12**	119871	128429	8557	+	23	99	**ALT RNA**	4.30*	N/D
**SJ-13**	128279	128429	149	+	359	223	**K14/ORF74**	-1.61	-1.54 (K14), -1.28 (ORF74)
**SJ-14**	18217	18094	122	-	21	4	**ORF2**	-5.26*	-2.60*(ORF2)
**SJ-15**	19696	19575	120	-	23	57	**ORF70/K3**	2.48	-2.67*(ORF70), -1.76* (K3)
**SJ-16**	19718	19575	142	-	31	36	**ORF70/K3**	1.16	-2.67*(ORF70), -1.76* (K3)
**SJ-17**	20050	19575	474	-	3393	2048	**ORF70/K3**	-1.67	-2.67*(ORF70), -1.76* (K3)
**SJ-18**	20379	19575	803	-	45	7	**ORF70/K3**	-6.25*	-2.67*(ORF70), -1.76* (K3)
**SJ-19**	20804	19575	1228	-	26	38	**ORF70/K3**	1.46	-2.67*(ORF70), -1.76* (K3)
**SJ-20**	20379	20263	115	-	13	13	**ORF70/K3**	1.00	-2.67*(ORF70), -1.76* (K3)
**SJ-21**	20804	20263	540	-	691	1839	**ORF70/K3**	2.66*	-2.67*(ORF70), -1.76* (K3)
**SJ-22**	22790	22043	746	-	12	2	**K4.2/K4**	-5.88	-1.80* (K4.2) -1.03 (K4)
**SJ-23**	26260	26091	168	-	625	511	**K5**	-1.22	-1.14 (K5)
**SJ-24**	26298	26091	206	-	1622	1232	**K5**	-1.32	-1.14 (K5)
**SJ-25**	26447	26091	355	-	11	4	**K6/K5**	-2.78	-1.97* (K6), -1.14 (K5)
**SJ-26**	27110	26091	1018	-	64	65	**K6/K5**	1.02	-1.97* (K6), -1.14 (K5)
**SJ-27**	27126	26091	1034	-	96	145	**K6/K5**	1.51	-1.97* (K6), -1.14 (K5)
**SJ-28**	27191	26091	1099	-	139	175	**K6/K5**	1.26	-1.97* (K6), -1.14 (K5)
**SJ-29**	27233	26091	1141	-	146	215	**K6/K5**	1.47	-1.97* (K6), -1.14 (K5)
**SJ-30**	27709	26091	1617	-	15	19	**K6/K5**	1.27	-1.97* (K6), -1.14 (K5)
**SJ-31**	27126	27036	89	-	74	15	**K6**	-5.00*	-1.97* (K6)
**SJ-32**	27191	27036	154	-	16	3	**K6**	-5.26	-1.97* (K6)
**SJ-33**	27233	27036	196	-	14	3	**K6**	-4.76*	-1.97* (K6)
**SJ-34**	69684	42544	27139	-	17	34	**ORF48/ORF25**	2.00	1.51* (ORF48), -1.13 (ORF25)
**SJ-35**	69684	50027	19656	-	64	107	**ORF48/ORF29**	1.67	1.51* (ORF48), 1.67* (ORF29b)
**SJ-36**	69270	67968	1301	-	10	36	**ORF47/ORF45**	3.60	-1.57 (ORF47), -1.33 (ORF45)
**SJ-37**	68506	68119	386	-	64	12	**ORF46/ORF45**	-5.26	-1.77* (ORF46), -1.33 (ORF45)
**SJ-38**	69096	68119	976	-	260	500	**ORF47/ORF45**	1.92	-1.56 (ORF47), -1.33 (ORF45)
**SJ-39**	69136	68119	1016	-	13	31	**ORF47/ORF45**	2.38	-1.56 (ORF47), -1.33 (ORF45)
**SJ-40**	69270	68119	1150	-	231	833	**ORF47/ORF45**	3.61*	-1.56 (ORF47), -1.33 (ORF45)
**SJ-41**	69684	68119	1564	-	75	333	**ORF48/ORF45**	4.44*	1.51* (ORF48), -1.33 (ORF45)
**SJ-42**	87967	87865	101	-	54	25	**K10 (vIRF4)**	-2.17	-1.18 K10 (vIRF4)
**SJ-43**	88558	88321	236	-	24	15	**K10 (vIRF4)**	-1.59	-1.18 K10 (vIRF4)
**SJ-44**	90463	90368	94	-	67	80	**K10.5 (vIRF3)**	1.19	-1.21 K10.5 (vIRF3)
**SJ-45**	93163	93041	121	-	37	47	**K11 (vIRF2)**	1.27	-1.30 K11 (vIRF2)
**SJ-46**	117854	117790	63	-	13	15	**K12**	1.15	1.61* (K12)
**SJ-47**	118145	117994	150	-	36	71	**ORF72/ORF71**	1.97	-1.31* (ORF72), -1.35* (ORF71)
**SJ-48**	122790	117994	4795	-	96	59	**ORF72/ORF71**	-1.64	-1.31* (ORF72), -1.35* (ORF71)
**SJ-49**	126873	122971	3901	-	27	23	**ORF73/ORF72/ORF71**	-1.18	-1.31 (ORF73), -1.31* (ORF72), -1.35* (ORF71)
**SJ-50**	126873	126373	499	-	31	17	**ORF73/ORF72/ORF71**	-1.82	-1.31 (ORF73), -1.31* (ORF72), -1.35* (ORF71)
**SJ-51**	134285	134199	85	-	20	13	**K15**	-1.54	1.10 (K15)
**SJ-52**	134469	134386	82	-	18	7	**K15**	-2.56	1.10 (K15)
**SJ-53**	134659	134576	82	-	14	16	**K15**	1.14	1.10 (K15)
**SJ-54**	134889	134811	77	-	34	51	**K15**	1.50	1.10 (K15)
**SJ-55**	135067	134978	88	-	29	36	**K15**	1.24	1.10 (K15)
**SJ-56**	135393	135309	83	-	19	51	**K15**	2.68	1.10 (K15)
**SJ-57**	135595	135485	109	-	12	28	**K15**	2.33*	1.10 (K15)

### KSHV RNA splice sites and their usage in viral RNA splicing

During RNA splicing, the intron recognition is mediated by binding of U1 snRNP to 5′ ss and U2 snRNP to 3′ ss. The binding affinity of these factors depends on the sequences surrounding the individual splice sites. Thus, analysis of splice site surrounding sequences can be used to determine the strength of each splice site and ultimately the efficiency of intron removal. We found that viral RNA splicing in the WT KSHV genome is mediated by a total of 187 distinct 5′ ss and 138 3′ ss. The identified 57 RNA splice junctions supported by ≥10 splice junction reads in the WT genome were found being mediated by splicing from 47 distinct 5′ ss to 35 3′ ss (**Tables 1 and 2**). The introns within the splice junctions are all canonical “GU-AG” introns. We found that most viral 5′ ss participate in splicing of a single intron. Only 9 (19%) of all 47 WT 5′ ss use an alternative 3′ ss, with the 5′ ss at nt 69,684 located in the ORF47/48 intergenic region being spliced mostly to three alternative 3′ ss (**Fig 4 and Table 2**). Similarly, only 8 (23%) of all 35 WT 3′ ss are associated with an alternative 5′ ss usage. A striking exception is that the 3′ ss at nt 26,091 upstream of K5 could accept 8 different 5′ ss for various RNA splicing events (**Fig 4 and Table 2**).

**Table 2:** The sequence and strength of identified KSHV splice sites. The position and sequences of KSHV 5′ (A) and 3′ (B) splice sites (SS) of splice junction (JS) with ≥10 splice reads. The numbered splice junctions (SJ) are shown in **Figs 3, 4 and 6** (see **Tables 1 and 3** for more details). In green color are the numbered splice junctions with ≥10 reads identified only in the 57KO genome. The strength (colored gradient) of individual SS was determined by MAX:ENT model [32].

A					B				
5′ SS					3′ SS				
nt	Strand	Sequence	Numbered SJ	MAX ENT	nt	Strand	Sequence	Numbered SJ	MAX ENT
981	+	CAGguaaga	1	10.77	2625	+	uccugccauuucuuuaacagAUU	1	10.22
28353	+	AUUgugggu	58,59	0.62	28903	+	ugguguguuuauuuuuccagUGU	58	7.74
61179	+	GAGgugaga	2	7.66	29112	+	cuaacgauguuuucuuguagGUG	59	8.79
63074	+	CAGguucgu	60	7.97	61307	+	uccuauguguuuauuuuuagCAA	2	8.67
69364	+	AUGguaagg	3, 61	9.33	64398	+	uaagaauacuugccuugcagGAU	60	6.13
71135	+	AAGguaaag	4	9.06	72094	+	acacgccacucucuccuuagGGU	3,4,62	11.38
71847	+	CAGguacgg	62	10.88	74993	+	cuguuuuugucucuuuaaagGCC	5,61	10.90
74845	+	AAGguaggg	5	8.76	75167	+	ccaccuugcugucuuuguagGCA	6	9.20
75085	+	CAGguauag	6	8.73	75955	+	acguucugucucaucuacagGAU	7,8,63	11.13
75360	+	GUGguaaga	7	8.24	81749	+	uaucgcguuugauauuacagACU	9	5.90
75677	+	CGAgugagu	63	8.49	83069	+	cuuuuauccacuuucuuaagGAU	10	7.19
75860	+	CAGguguau	8	3.09	92033	+	auagccuguuucuuacucagGUA	64	7.85
81640	+	AGGguaagu	9	10.45	93358	+	guuucauuuuucccuuauagAUG	65	10.99
82497	+	UGGgugagu	10	8.73	127529	+	acucauuugguggccggcagGUG	66	6.95
91845	+	GAAguaagc	64	6.95	127583	+	ccccuacuggacacgugcagGUA	11	4.03
93066	+	AAGguaagu	65	11.00	128429	+	uauacuacuuguuauuguagGCC	12,13,67,68,69	6.44
119871	+	GAGguagga	11,12,66	8.24	18094	-	guguuucucucgcaugauagCUU	14	8.47
120239	+	UUAguaagu	67	7.79	19575	-	uauuguggguucucucucagGAU	15,16,17,18,19	8.02
127582	+	CAGguaaau	68	8.76	20263	-	gcucauguuacuggucuuagACC	20,21,70	3.51
127766	+	CAAgugagg	69	5.85	22043	-	uuugucugguuaccuugcagGUU	22	10.20
128279	+	UAGgugggu	13	5.56	26091	-	cuccccuuucccuuuuucagACU	23,24,25,26,27,28,29,30	11.81
18217	-	AAGguuugg	14	5.28	26446	-	gcgugucccuuauuucauagGUC	71	10.13
19696	-	CCAguaggu	15	5.41	27036	-	gccuuuacgguuuucuuuagACU	31,32,33	7.49
19718	-	ACAgugagu	16	8.34	42544	-	ccaucuaucauucuccgcagGUC	34	11.52
20050	-	CAGguaggg	17	9.46	50027	-	gugucuuuccgugucugcagAGC	35	10.73
20379	-	ACGgugagc	18,20	9.44	67968	-	accucuuucaucacuuccagGAU	36,72,73	11.43
20804	-	CAGguauag	19,21	8.73	68119	-	uuguaaauuuccgccccuagCGG	37,38,39,40,41	8.08
20935	-	GAGguaacu	70	8.77	71414	-	ccuuuguuuuuuuuggacagCUG	74	8.71
22790	-	CUGguguuu	22	-2.10	73240	-	agcgaccugaucucuugcagGAG	75,76,77	7.35
26260	-	AGGgugggu	23	5.37	87865	-	cucucgugccuuuuacuuagAGA	42	6.62
26298	-	CCUgugcgu	24	4.27	88321	-	gaacaaccuucuuuuugcagCCC	43	7.86
26447	-	UAGguccgg	25	0.31	90368	-	auuuguuuuuuuuucuguagGCC	44	10.78
27110	-	CAGguaagc	26	9.88	93041	-	uguaauuaacuuuuguuuagGGA	45	6.22
27126	-	CAGgugaga	27,31	9.22	117790	-	gucccccccccgcaccccagGAA	46	7.67
27191	-	UUGgugagu	28,32	9.27	117994	-	cgguuacgcccccuucgcagGAA	47,48	7.89
27233	-	UAAguaagc	29,33,71	5.66	122971	-	ucccccuuuuuccucccuagAAG	49	11.34
27709	-	CAGgugauu	30	5.95	126373	-	aauuuuacuuugguugucagACC	50	4.42
68506	-	AGGgugugu	37	4.51	134199	-	uuuucuauuuuaaaauuuagGUA	51	9.47
69096	-	ACUguaggu	38,72	4.15	134386	-	aacuauccucuauuuuuuagCGU	52	8.23
69136	-	GAGguuguu	39	0.25	134576	-	guguuuuuauguauaaacagGCC	53	8.78
69270	-	GGGgucugu	36,40	0.78	134811	-	guuaacgacauuuuuuguagGAU	54	5.66
69684	-	CAGguaaug	34,35,41,73	9.43	134978	-	ucgguuaucuuuuuuuauagGCU	55	8.40
71509	-	GUGguaaaa	74	2.98	135309	-	auucuuuuuguauuuuauagCUU	56	9.26
73404	-	CAGguaacc	75	8.66	135485	-	acauacauuuguuuuuauagCUU	57	7.94
73817	-	GGGguaacg	76	4.76					
74027	-	AAGgugacg	77	7.00					
87967	-	UAGguaugu	42	7.76					
88558	-	UAGgugagu	43	8.83					
90463	-	AAGguaaga	44	10.57					
93163	-	GAGguagga	45	8.24					
117854	-	CAGguagua	46	5.89					
118145	-	CUAgugagu	47	7.43					
122790	-	CAGguaggc	48	9.88					
126873	-	ACAgugagu	49,50	8.34					
134285	-	UCAguaagu	51	9.14					
134469	-	CAGguaggu	52	10.28					
134659	-	AAGguuugu	53	7.81					
134889	-	UGGguacag	54	0.89					
135067	-	CAGguaagu	55	10.86					
135393	-	CAGguaggu	56	10.28					
135595	-	UUGguaagu	57	10.47					

To understand the contribution of splice sites to KSHV RNA splicing regulation, we analyze their strength by a maximum entropy (MAX:ENT) model based on sequence composition surrounding 5′ ss and 3′ ss [32]. As shown in **Table 2,** most WT 5′ ss represent the optimal 5′ ss with a median MAX:ENT 8.24, and only six 5′ ss have a suboptimal MAX:ENT below 4.0 (**Table 2A**). While most suboptimal intron 5′ ss contribute to relatively low-frequency splicing, two of these splice sites mediate RNA splicing to express functional K8.1 (SJ-8) and K15 (SJ-54) proteins. Their relative weakness could be overcome by their optimal exon size of the upstream exons (K8.1, K15) and presumably the stimulatory effect by splicing of adjacent introns (K15) [33]. Like 5′ ss, most WT KSHV 3′ ss are relatively strong, with a median MAX:ENT 8.32 (**Table 2B**). Interestingly, the only weak 3′ ss with MAX:ENT under 4.0 was mapped at nt 20,263 within the ORF70 coding region and the splicing of this intron in the ORF70 coding region subjects to ORF57 regulation (see our data in the latter text). Another relatively weak 3′ ss (MAX:ENT 4.42) was found in the LANA 5′ UTR at nt 126,373 and could accept a 5′ ss at nt 126,873 (SJ-50) for LANA RNA splicing. Interestingly, the same 5′ ss is also spliced to a much stronger (MAX:ENT 11.34), but distal 3′ ss at nt 122,971 upstream of ORF72 encoding vCyclin (SJ-49) [16]. Both RNA splicing events in the polycistronic LANA RNA occur in comparable frequency, indicating that the relative weakness of the proximal 3′ ss in LANA RNA may allow splicing of this 5′ ss to a more distal strong 3′ ss in order to facilitate the expression of all latent proteins from this polycistronic RNA transcribed from the single latent constitutive promoter [15].

### KSHV RNA splicing profile from the 57KO genome in BCBL-1 cells

**Fig 6:**
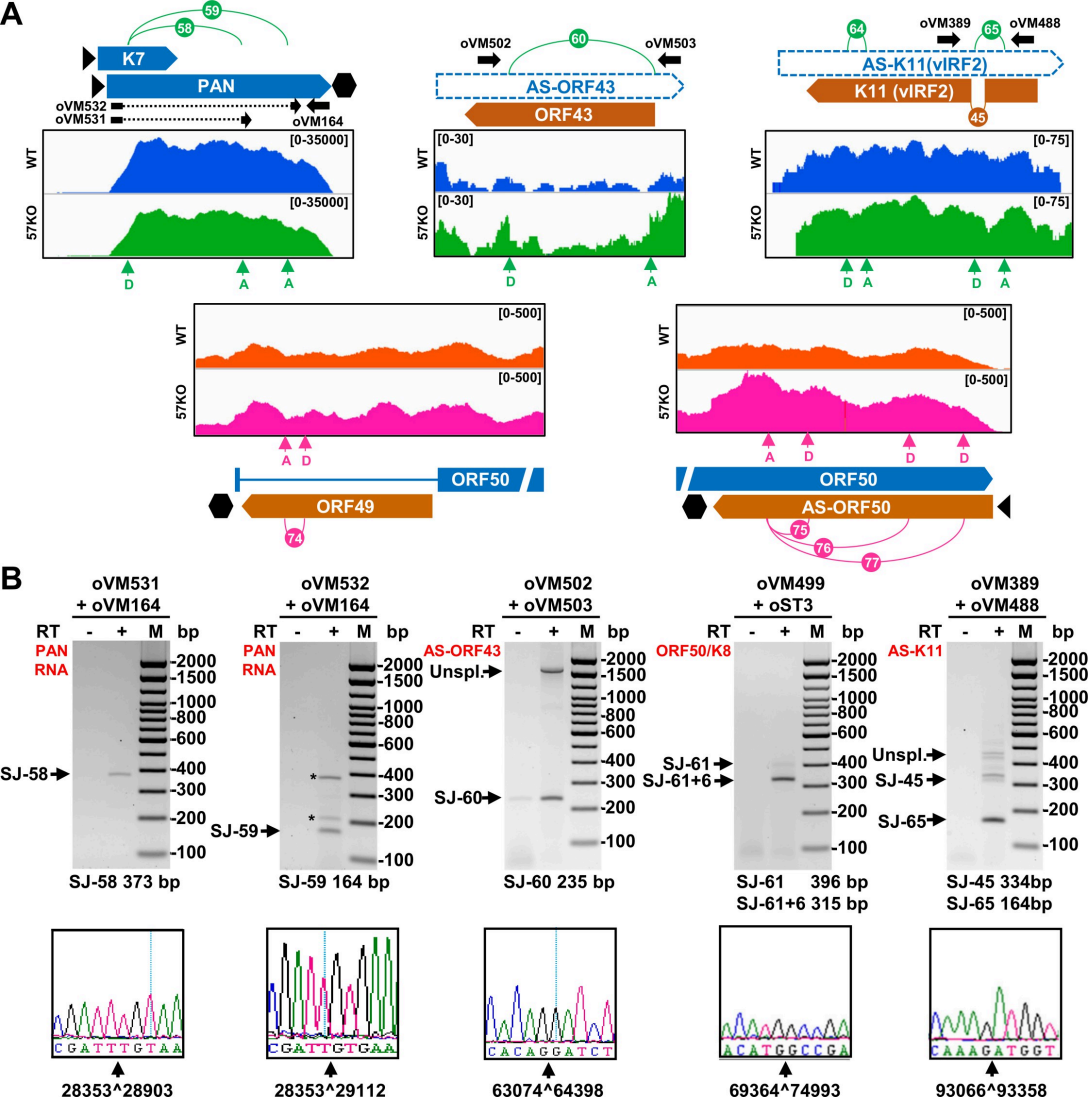
Mapping of 57KO-specific splice junctions in intronless and antisense viral transcripts. (A) The RNA splice junctions supported with ≥10 splice reads from the 57KO genome were mapped to intronless viral transcripts. See **Figs 3** and **4** for a detailed description. (B) RT-PCR validation of selected 57KO-specific RNA splicing events using total RNA from BCBL-1 57KO cells treated with VA for 24 h. The sizes of expected amplicons with indicated primer pairs in the presence of reverse transcriptase (+RT) are shown below, together with the chromatographs from Sanger sequencing confirming the mapped splice junctions. The asterisks (*) mark the non-specific RT-PCR amplicons. SJ-61+6 means a RT-PCR product from the double RNA splicing first from the splice junction SJ-61 and then SJ-6 (see **Fig 3**).

Similarly, we analyzed the biological significance of viral splice junctions detected in viral RNAs from the 57KO genome in BCBL-1 cells. As mentioned above, we identified 70 viral splice junctions with ≥ 10 splice junction reads from the 57KO genome, of which 50 were also observed from the WT KSHV genome (**Fig 2B**). The additional 20 splice junctions (SJ-58 to SJ-77) with ≥ 10 splice junction reads were found in the 57KO genome, but less than 10 splice junction reads in the WT KSHV genome (**Table 3**). These RNA splice junctions could be split into two groups based on their mapping. The first group represents splice junctions mapped to viral transcripts with notable splice sites in the WT genome (**Figs 3 and 4**), but these splice sites could be alternatively spliced to another splice site in the 57KO genome, thus creating a new splice junction enriched in the 57KO genome. This group of viral RNA transcripts includes the alternatively spliced RNAs from ORF50/K8/K8.1 (SJ-61 to -63), ALT RNA (SJ-66 and -67), K14/ORF74 (SJ-68 and -69), ORF70/K3 (SJ-70), K5/K6 (SJ-71), and ORF45/46/47/48 (SJ-72 and -73) (**Figs** 3, 4 **and** S2A). The second group represents nine novel splicing events not reported or documented in the KSHV intronless RNA transcripts. They were detected from viral lncRNA PAN (SJ-58 and -59) and antisense RNA to ORF43 (SJ-60), antisense RNA to K11 (SJ-64 and -65), ORF49 (SJ-74), and antisense RNA to ORF50 (SJ-75 to -77) (**Fig 6A**). These splice junction reads could be readily detected and verified by RT-PCR and sequencing (**Fig 6B**). It is worth noting that most of these RNA splice junctions could also be detected from the WT genome with less than 10 splice junction reads (**S2B Fig and Table 3**), but the SJ-68 was found only from the 57KO genome. Although detected in the expression of both 57KO and WT genomes, the spliced PAN RNA was just a tiny fraction among the abundant PAN transcripts. We identified total 54 RNA splice junction reads in the WT cells out of total 3,307,738 reads mapped to the PAN locus (**S1 and S2 Tables**). Similarly, we identified total 86 splice junction reads out of total 2,057,347 reads mapped to PAN in the 57KO cells. Thus, the splice junction reads from the PAN region represent only 0.0016% of the WT and 0.0042% of the 57KO total PAN reads. Nevertheless, these data indicate that loss of ORF57 expression led to alternative splicing of KSHV RNAs by activation of cryptic splice site usage in the intron-containing and/or intronless viral transcripts.

**Table 3:** The additional splice junctions (SJ) with ≥10 splice reads detected predominantly from the 57KO genome in BCBL-1 cells. For details, see Table 1.

Splice junction	5′ SS (nt position)	3′ SS (nt position)	Intron size (nt)	Strand	Reads		Transcript	SJ FC 57KO/WT	FC 57KO/WT
					WT	57KO			
**SJ-58**	28353	28903	549	+	6	30	**K7/PAN**	5.00	-1.33* (K7), -1.34 *(PAN)
**SJ-59**	28353	29112	758	+	8	26	**K7/PAN**	3.25	-1.33* (K7), -1.34 *(PAN)
**SJ-60**	63074	64398	1323	+	2	40	**AS-ORF43**	20.00	N/D
**SJ-61**	69364	74993	5628	+	6	11	**ORF50/K8/K8.1**	1.83	1.30 (ORF50), 1.09 (K8), 1.03 (K8.1)
**SJ-62**	71847	72094	246	+	8	43	**ORF50/K8/K8.1**	5.38	1.30 (ORF50), 1.09 (K8), 1.03 (K8.1)
**SJ-63**	75677	75955	277	+	8	13	**ORF50/K8/K8.1**	1.63	1.30 (ORF50), 1.09 (K8), 1.03 (K8.1)
**SJ-64**	91845	92033	187	+	3	10	**AS-K11 (vIRF2)**	3.33	N/D
**SJ-65**	93066	93358	291	+	6	26	**AS-K11 (vIRF2)**	4.33	N/D
**SJ-66**	119871	127529	7657	+	8	11	**ALT RNA**	1.38	N/D
**SJ-67**	120239	128429	8189	+	7	52	**ALT RNA**	7.43	N/D
**SJ-68**	127582	128429	846	+	0	12	**K14/ORF74**	N/A	-1.54 (K14), -1.28 (ORF74)
**SJ-69**	127766	128429	662	+	1	17	**K14/ORF74**	17.00	-1.54 (K14), -1.28 (ORF74)
**SJ-70**	20935	20263	671	-	7	11	**ORF70/K3**	1.57	-2.67*(ORF70), -1.76* (K3)
**SJ-71**	27233	26446	786	-	7	22	**K6/K5**	3.14	-1.97* (K6), -1.14 (K5)
**SJ-72**	69096	67968	1127	-	6	13	**ORF47/ORF45**	2.17	-1.56 (ORF47), -1.31 (ORF45)
**SJ-73**	69684	67968	1715	-	3	12	**ORF48/ORF45**	4.00*	1.51* (ORF48), -1.31 (ORF45)
**SJ-74**	71509	71414	94	-	4	14	**ORF49**	3.50	1.31 (ORF49)
**SJ-75**	73404	73240	163	-	4	19	**AS-ORF50**	4.75*	N/D
**SJ-76**	73817	73240	576	-	6	10	**AS-ORF50**	1.67	N/D
**SJ-77**	74027	73240	786	-	5	11	**AS-ORF50**	2.20	N/D

We previously identified ORF57 protein as a viral splicing factor promoting splicing of K8 intron 2 to express functional K8α [17] by interaction with host splicing factor SRSF3 to attenuate its suppressive activity on K8 intron 2 splicing [18]. Surprisingly, we did not observe an obvious retention of K8 intron 2 from the 57KO genome over the WT genome (**Fig 3 and Table 1**). Although the cause of this discrepancy remains to be understood, we were assuming that the remaining expression of PAN RNA from the 57KO genome might compensate ORF57 loss by sponging SRSF3 in the 57KO cells. As expected, we found that overexpression of PAN RNA could promote K8 intron 2 splicing in HEK293T cells (**S3A Fig**). Additional reduction of SRSF3 RNA and protein levels due to virus-host shut off during lytic replication (**S3B Fig**) may further reduce the dependence of K8 splicing on ORF57, especially in the later stage of KSHV infection.

### Presence of the long-range RNA splicing events crossing over the KSHV genome

**Fig 7:**
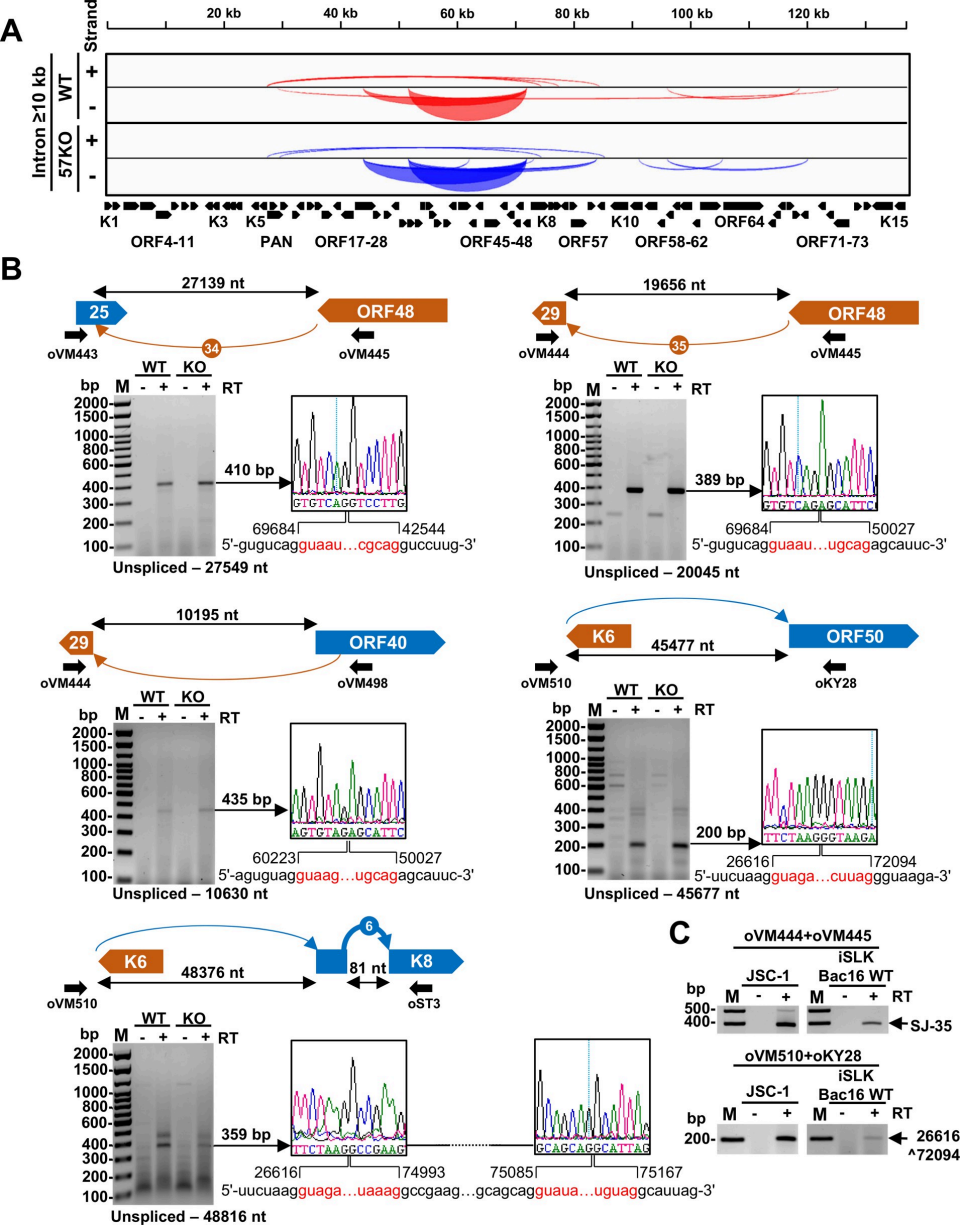
Long-range KSHV RNA splice identified in BCBL-1 cells with lytic KSHV infection. (A) The Sashimi plot showing all splice junctions with an intron size ≥10 kb supported with at least 2 splice junction reads. See **S2 Table** for details. (B) Validations of selected long-range RNA splicing events by RT-PCR on total RNA from the WT genome or 57KO genome in the cells with lytic KSHV replication induced by VA for 24 h. Above the gel is a schematic showing a selected splicing event in orange arches for minus DNA strand-derived RNA transcripts and blue arches for plus DNA strand-derived RNA transcripts, relative genomic locations, predicted intron sizes, and primers used for RT-PCR. The arches lacking number-based nomenclature did not pass ≥10 of supporting splice reads (see **S2 Table**). Below is the gel electrograph of obtained RT-PCR products. The obtained RT-PCR products were reamplified and confirmed by Sanger sequencing. See sequencing primers in **S6 Table**. The chromatographs on the right show the identified splice junctions with the exonic sequences marked in black and the intronic sequences spliced out shown in red. The numbers represent exon-exon junction positions in the KSHV genome (GenBank Acc. No. HQ404500) after RNA splicing. (C) The conservation of long-range splicing events in JSC-1 and iSLK/Bac16 cells. Total RNA from the cells with induced KSHV lytic infection was used in RT-PCR using primers showed in (B). See **S4A Fig** for more details.

Most previously described RNA splice junctions represent the highly prevalent splice junctions supported by ≥10 splice junction reads, which were mapped within well-defined transcriptional units (**Figs 3, 4 and 6**). However, we also detected numerous long-range splicing events crossing over the large parts of the virus genome both from the WT and 57KO genomes (**Fig 2C**). To better define these splicing events, we first extracted all splice junction reads covering an intron size ≥10 kb and had at least two splice reads either from one genome or both WT and 57KO genomes (**Fig 7A**). We found a total of 14 such splice junctions, 7 of them from the WT genome and 10 from the 57KO genome, with 3 found consistently from both genomes in BCBL-1 cells (**S2 Table**). A small overlap between the WT genome and the 57KO genome suggests a possible role of ORF57 in regulation of generating these RNAs. Most long-range splicing events occur in low frequency with a few (less than 10) splice reads and only two long-range splice junctions derived from ORF48 splicing to ORF25 (SJ-34) and from ORF48 splicing to the second exon of ORF29 (SJ-35) passed ≥10 supporting splice reads threshold (**Fig 4 and Table 1**). All long-range splice junctions were generated using canonical splice sites (**S1 Table**). To verify selected long-range splicing events, we performed RT-PCR using various sets of flanking primers on total RNA isolated from both WT and 57KO BCBL-1 cells. Sanger sequencing of the obtained RT-PCR products confirmed the mapped splice junctions (**Fig 7B**). Some of these long-range RNA splicing events were also detectable in iSLK/Bac16 and JSC-1 cells with lytic KSHV infection (**Figs 7C and S4A**). Together, these data indicate the existence of numerous conserved long-range splicing events in KSHV RNA processing. Although their biological relevance remains to be determined, these long-range spliced RNA isoforms may encode several new proteins (**S4B Fig**) lacking any homology to the known viral proteins worth further investigating in future studies.

### Genome-wide KSHV RNA splicing and circular RNA production

**Fig 8:**
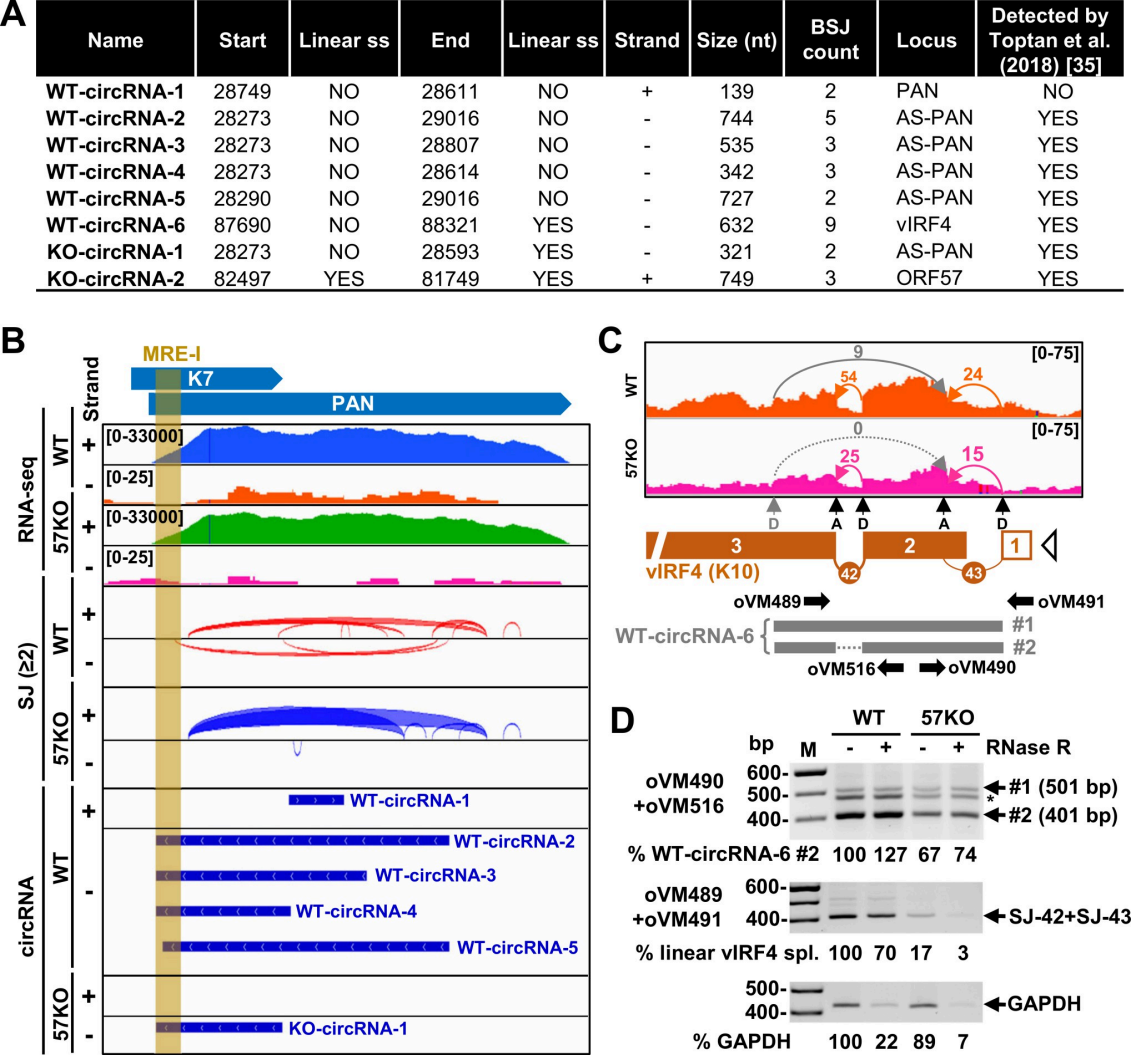
Linear RNA splicing and production of KSHV circRNAs. (A) Identification of KSHV circRNAs from RNA-seq in BCBL-1 cells with a WT or 57KO genome during lytic replication induced by 24 h valproic acid treatment using a circular RNA prediction software CIRI2 [38, 39]. (B) Distribution and coverage of RNA-seq reads on PAN, detected linear PAN RNA splice junctions (red and blue arches) with minimum 2 splice junction reads and predicted viral PAN circRNAs (thick blue lines, see panel A for details) in WT and 57KO BCBL-1 cells with lytic replication. MRE-I represents the Mta or ORF57-responsive element I in PAN RNA identified by anti-ORF57 CLIP [26]. (C) The RNA-seq reads mapped to KSHV vIRF4 (K10) locus in WT (orange) and 57KO (pink) cells with reads-coverage depth shown in the upper right corner. The arches show a direction and number of splice reads detected for linear canonical splicing (orange and pink arches) and back-splicing (grey arches). Below is a diagram of KSHV vIRF4 locus with detected exons (orange boxes), introns (numbered orange arches) with the corresponding splice sites (D = splice donor and A = splice acceptor). The open triangle represents a predicted transcriptional start site (TSS). Predicted unspliced and spliced forms of WT-circRNA-6 are shown in grey. The primers used to amplify linear and circular vIRF4 RNAs are shown as arrows. (D) The effect of 57KO on the production of vIRF4 linear and circRNAs. The total RNA from WT or 57KO cells isolated 24 h after induction of viral lytic cycle with or without RNase R digestion was used as a template to amplify circular vIRF4 RNA using a primer pair of oVM490 and oVM516 and linear spliced vIRF4 RNA using a primer pair of oVM489 and oVM491 as shown in the panel C. Host GAPDH RNA was amplified as an RNA loading control with the primer pair shown in the **S6 Table**. The percentage of individual RNA was calculated based on band signal density using ethidium bromide-stained agarose gels using ImageLab software (BioRad) with the amount of PCR products obtained from WT RNA without RNase R treatment set as 100%. The asterisk (*) indicates a heteroduplex band derived from two RT-PCR products as described [75].

RNA splicing usually occurs from the 5′ to 3′ direction and produces a spliced RNA in linear form. However, RNA splicing may occasionally generate a circular RNA (circRNA) by back-splicing when a downstream 5′ ss splices back to the upstream 3′ ss crossing over the exon(s) [34]. Viral circRNAs were reported in KSHV-infected cells; many of them were generated from the RNA transcripts lacking any RNA splicing [35–37]. We subsequently performed viral circRNA prediction from the same RNA-seq library reads using CircRNA Identifier CIRI2 pipeline (https://sourceforge.net/projects/ciri/) [38,39] (**S3 Table**). As shown in **Fig 8A**, we predicted a total of eight KSHV viral circRNAs, of which 6 were from the WT and 2 from the 57KO genome. Among 6 circRNAs from the WT genome, one mapped to PAN, four to antisense PAN (also referred to as K7.3), and one to vIRF4 (K10). Only 2 viral circRNAs were predicted from the 57KO genome, with one mapped to the antisense PAN and the other to ORF57. The supporting back-splicing reads to individual circRNAs were relatively low, ranging from 2 to 9 back-splicing reads from all samples in each group. All viral circRNAs, except WT-circRNA-1, were predicted in low abundance in BCBL-1 cells by Toptan and colleagues [35]. Given the circRNA being generated by back-splicing from the splice sites used for linear RNA splicing, we next compared all predicted circRNAs in production coordination with those splice sites supported by at least two splice junction reads identified in our study (**S4 Table**). We found that most viral circRNAs (**Fig 8A and 8B**) are not supported by the splice sites used for linear RNA splicing, besides WT-circRNA-6 and KO-circRNA-2. As the ORF57 gene is reversely orientated in the 57KO genome [21], the KO-circRNA-2 was genuinely back-spliced by crossing over the exon 2 [40]. We subsequently performed the RT-PCR using several inverse primer pairs on total RNA from the WT and 57KO genome in BCBL-1 cells with lytic induction to verify the predicted viral circRNAs. Unfortunately, we failed to detect any circRNAs from the PAN region even using 40 amplification cycles and 500 ng of cDNA derived from total RNA extracted either from BCBL-1 cells with a WT or 57KO genome. Even we could not verify any; it is worth noting that the reported PAN circRNAs [35–37] appear mainly next to the ORF57-MRE-I motif we previously identified for PAN RNA stability [26] (**Fig 8B**). We noticed that the predicted PAN circRNAs from Toptan’s report were at different positions from the reported PAN circRNAs in Tagawa’s report (**S5 Fig**) [35, 36]. Ungerleider’s report had no description of PAN circRNAs at all [37]. Subsequently, we analyzed all reported PAN circRNAs with more than 1000 back-splicing junction reads along with our predicted PAN circRNAs for their splice site strength (**S5 Fig** and **S5 Table**). We found none of them give a reasonable splice site score strong enough to carry out a true RNA splicing reaction (**S5 Fig**). Data suggest that all predicted PAN circRNAs with the current prediction program were unreal.

Interestingly, the predicted WT-circRNA-6 matches the previously identified circRNA from vIRF4 RNA [35,37]. We identified two linear RNA splicing events in vIRF4, SJ-42, and -43, with SJ-42 reads being about twice more abundant than SJ-43 (**Fig 8C and Table 1**). It is worth noting that retention of the SJ-43 intron is required to express full-length vIRF4 protein. The 3′ ss of the SJ-43 intron in vIRF4 also functions as an acceptor site for the generation of WT-circRNA-6, whereas the donor site used for WT-circRNA-6 production is in vIRF4 exon 3 and not even used for RNA splicing of linear vIRF4 RNA. As a result, the second intron could be spliced or retained in vIRF4 circRNAs, leading to the generation of two isoforms of circRNA (**Fig 8C**). We observed about two-fold reduction of splice junction reads in BCBL-1 cells with the 57KO genome when compared with the WT. Using different primer sets in RT-PCR and RNase R digestion, we were able to detect and distinguish the linear from circular vIRF4 RNAs and the reduced expression of vIRF4 RNA from the 57KO genome in BCBL-1 cells when compared with the WT genome (**Fig 8D**), where the linear vIRF4 RNA in the 57 KO cells appeared ~80% reduction and accompanied by ~20% reduction of the vIRF4 circRNA. Compared to GAPDH, we also found the linear vIRF4 RNA appeared unexpectedly a little more resistant to RNase R digestion (**Fig 8D**).

### Effect of ORF57 on global KSHV RNA splicing

To fully comprehend the contribution of ORF57 to KSHV RNA splicing regulation, we also compared the number of splice reads assigned to each splice junction detected from both WT and 57KO genomes (see **Table 1**). Using a minimal 2-fold change cut-off, we identified 21 dysregulated RNA splicing events from the 57KO genome, with 11 being downregulated and 10 being upregulated. As expected, the most downregulated was the splicing of ORF57 first intron (SJ-9) due to the inversed ORF57 orientation in the 57KO genome [21]. Other downregulated splicing events due to lack of ORF57 expression were mapped to RNA transcripts from ORF2 (SJ-14), ORF70/K3 (SJ-18), K4.2/K4 (SJ-22), K5/K6 (SJ-25, -31, -32, and -33), ORF45/46 (SJ-37), K10 (SJ-42), and K15 (SJ-52). The RNA splicing events upregulated in the 57KO cells, ranging from 2.00- to 4.44-fold, were mapped to the transcripts of ALT RNA (SJ-12), ORF70/K3 (SJ-15 and -21), ORF48/ORF25 (SJ-34), ORF47/ORF45 (SJ-36, -39, -40), ORF48/ORF45 (SJ-41), and K15 (SJ-56 and -57). Even though the expression of most KSHV genes is not significantly altered from the 57KO genome, some of the observed splicing changes resulted in altered gene expression. A downregulation of ORF2, K6, and K4.2/K4 expression in the 57KO cells may at least partially explain a reduction of splice junction reads in these transcripts. On the contrary, the splicing of some transcripts, like ORF47/45, was increased from the 57KO genome despite their overall transcription level was reduced (**Table 1**). Together, this study discovered that ORF57 has both stimulatory and suppressive effects on KSHV RNA splicing, which could also be confirmed by RT-PCR (**S2C Fig**). Subsequently, we focused on the altered RNA splicing of KSHV K15 and ORF70/K3, two viral transcripts containing multiple introns and expressed from two different genomic loci without overlapping transcription. Thus, we could examine how ORF57 directly affects alternative RNA splicing of these two different transcripts.

### KSHV ORF57 regulates alternative RNA splicing of K15

**Fig 9:**
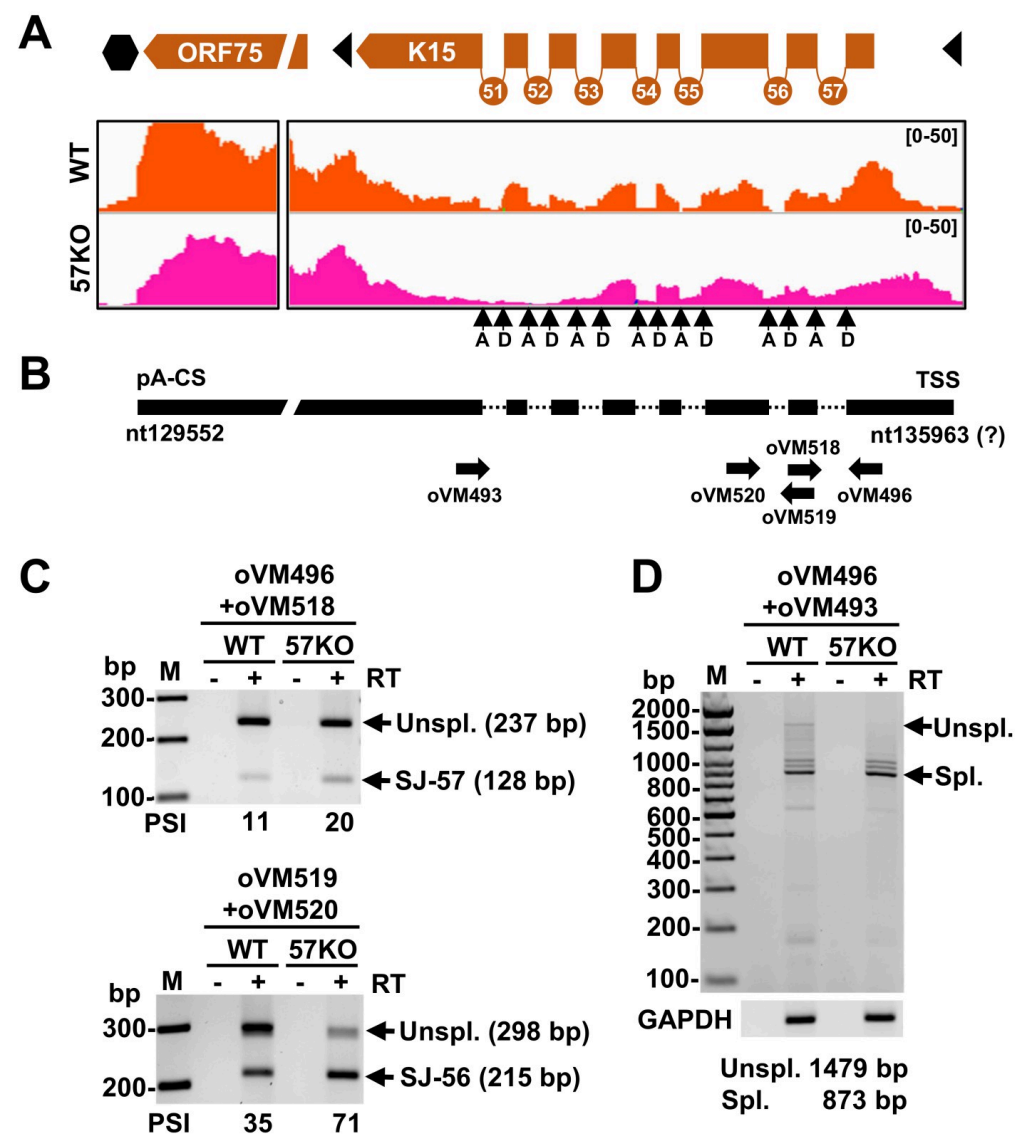
ORF57 regulates K15 RNA alternative splicing. (A) The splice junctions and RNA-seq coverage of K15 transcripts detected in BCBL-1 cells bearing a WT (orange) or 57KO genome (pink) during KSHV lytic infection. Black arrows mark splice donor (D) and acceptor (A) sites. (B) Structure of the K15 pre-mRNA with the position of mapped pA cleavage site (CS) and predicted transcriptional start site (TSS). The thick black lines represent the exons and the dotted lines the introns. The arrows below show the positions and orientations of the primers used in RT-PCR listed in the **S6 Table**. (C and D) Gel electrographs of RT-PCR products obtained from total RNA from the WT and 57KO genomes in BCBL-1 cells undergoing lytic replication harvested 24 h after VA treatment, with the primer pairs shown in (B). The samples without reverse transcription (- RT) were used as a negative control. The host GAPDH was used as a loading RNA control. Unspl., unspliced pre-mRNA; Spl., spliced mRNA; PSI, percent spliced-in of the alternative exon (s) or splice site (% inclusion = inclusion/sum of inclusion + exclusion) [76].

K15, located at the right end of the long unique region of the KSHV genome, encodes a viral membrane protein with a variable number of transmembrane domains [41,42]. Through its cytoplasmic C-terminus containing one proline-rich Src homology 3 (SH3) and two SH2-binding sites, K15 interacts with several host factors, such as phospholipase Cγ1 (PLC γ1) to activate numerous cellular signaling pathways [43]. K15 is expressed in both latent and lytic infected cells. During latency, K15 promotes the invasiveness of KSHV tumors, KSHV-induced angiogenesis, and the survival advantage of infected cells [44,45]. By activating PLC γ1, ERK1/2 and AKT1 signaling pathways, K15 promotes reactivation of KSHV from latency and is required for KSHV lytic replication [46,47]. K15 pre-mRNA harbors eight exons and seven short introns (**Fig 9A**). All internal introns are required to express K15 isoforms with a variable number of transmembrane domains via alternative splicing [41,42]. We found that the 57KO genome exhibited at least a 2-fold increase of its intron 1 (SJ-57) and intron 2 (SJ-56) splicing, but a substantial decrease (below 10 splice junction reads) of intron 6 (SJ-52) splicing (**Table 1**). Using the primer sets shown in **Fig 9B**, RT-PCR confirmed the increased intron 1 and intron 2 splicing in K15 RNA expressed from the 57KO versus the WT genome (**Fig 9C**). Calculated PSI (percentage spliced-in index) for both introns also showed a 2-fold increase of the splicing from the 57KO genome over the WT genome, as shown by RNA-seq analysis (**Table 1**). Consistently, we observed an increased amount of fully spliced K15 RNA expressed from the 57KO genome in BCBL-1 cells (**Fig 9D**). In contrast, expression of ORF57 from the WT genome led to an increased level of unspliced K15 RNA, accompanied by decreased level of fully spliced K15 RNA (**Fig 9D**). Retention of the intron 1 or intron 2 from K15 RNA splicing in the presence of ORF57 creates a premature termination codon in these isoforms of K15 RNA, presumably triggering the non-sense mediated RNA decay. We also observed a few other K15 introns with alternative RNA splicing in the WT or the 57KO genome, but none passed ≥10 reads threshold (**S2 Table**). More specifically, in the WT cells, we observed exon 5 skipping and use of an alternative 3′ ss in exon 8, whereas the cells containing the 57KO genome showed the usage of an alternative 5′ ss in exon 1 and an alternative 3′ ss in intron 1. Together, our data indicate that, by regulating K15 RNA splicing, ORF57 might alter the levels of full-length K15 protein and its isoforms during lytic infection.

### KSHV ORF57 promotes the RNA splicing-dependent expression from K3 to ORF70

**Fig 10:**
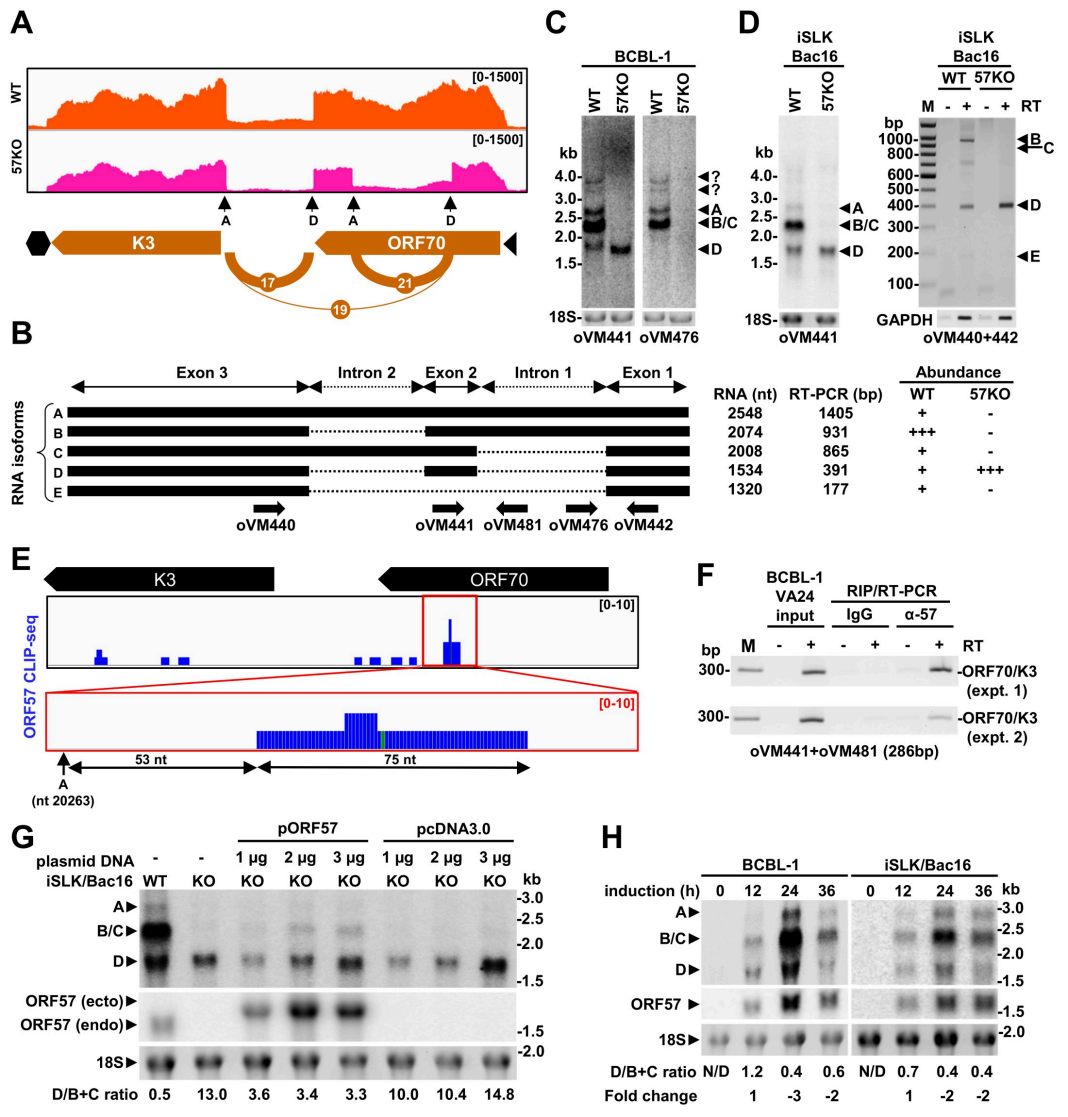
Regulation of ORF70/K3 RNA alternative splicing by ORF57. (A) The most prevalent splice junctions and RNA-seq reads-coverage of ORF70/K3 transcripts from the WT genome (orange) and the 57KO genome (pink) in BCBL-1 cells during KSHV lytic infection. (B) Diagrams of major ORF70/K3 splicing isoforms. Black boxes represent the exons and dashed lines the introns. The arrows below represent oligos used in Northern blot and RT-PCR (see **S6 Table** for the details). On the right are predicted sizes of all spliced isoforms and their relative abundance in the cells with the WT or 57KO genome. (C and D) Northern blot analysis of ORF70/K3 splicing isoforms expressed from the WT and 57KO genomes in BCBL-1 (C) and iSLK/Bac16 (D) cells with KSHV lytic replication. Total cell RNA was extracted and examined by Northern blot using two separate ^32^P-labeled oligo probes (oVM441 and oVM476) (C and D) or by RT-PCR using a primer set of oVM440 and oVM442 shown in (B) in the absence (-) or presence (+) of reverse transcriptase (RT) (D, right panel and **S2C Fig**). The 18S rRNA in Northern blot and GAPDH RNA in RT-PCR analyses indicate the amount of total RNA in individual sample loading. (E-F) ORF57 binding to ORF70/K3 RNA. (E) Distribution of ORF57 CLIP-seq reads (blue) mapped to the ORF70/K3 locus. The red box marks the zoomed-in area shown below. The ORF57 CLIP-seq was described in our previous report [53], and the obtained reads are publicly available in NCBI GEO (Acc. No. GSE179726). The intron 1 3′ ss (acceptor site) at nt 20263 is indicated as an arrow A. (F) The RNA-immunoprecipitation (RIP) assay was performed using whole-cell extracts from valproic acid (VA) treated BCBL-1 cells after UV crosslinking with an anti-ORF57 antibody or a control non-specific IgG. The pulled-down RNA was used as a template in RT-PCR to detect ORF70/K3 RNA with oVM441 and oVM481 primers (B). (G) Splicing rescuing of ORF70/K3 B isoform production from the 57KO genome by ectopic ORF57 expression. The iSLK/Bac16 cells with the 57KO genome were transfected with the increased amount of ORF57-expression vector (pORF57) or an empty vector (pcDNA3.0), followed by 24 h induction of lytic replication with DOX/Bu treatment. Total RNA from the transfected cells was isolated and analyzed by Northern blot together with the RNA from non-transfected cells with a WT and 57KO KSHV genome as controls. The oVM441 probe was used to detect the ORF70/K3 splicing isoform and oVM11 for detection of ectopic (ecto.) or endogenous (endo.) ORF57 expression. The 18S rRNA indicates the amount of total RNA in individual sample loading. (H) Correlation of ORF57 expression with ORF70/K3 isoform production during KSHV lytic replication. Total RNA isolated from BCBL-1 cells at various time points after induction of lytic replication by valproic acid (VA) was analyzed by Northern blot using ORF70/K3-specific probe oVM441 (B) and ORF57-specific probe (oVM11) (**S6 Table**). The 18S rRNA level detected by ethidium bromide staining was used as a loading control.

ORF70/K3, a bicistronic RNA with complex alternative splicing, is the second viral RNA of which splicing was notably affected from the 57KO genome (**Table 1**). It encodes viral thymidylate synthase ORF70 and viral E3-ubiquitin ligase K3 [48, 49]. We mapped two major introns in ORF70/K3 RNA as reported [6,50]. The 540 nt-long intron 1 (SJ-21) lies within the ORF70 coding region, while the 474 nt-long intron 2 (SJ-17) sits entirely into the intergenic region between ORF70 and K3 (**Fig 10A**). Similar to previous reports, we also observed low-frequent RNA splicing from the intron 1 donor site to the intron 2 acceptor site (SJ-19), resulting in removing a 1228 nt-long intron [6]. Several RNA isoforms generated from this region by alternative RNA splicing were reported in PEL cells with lytic KSHV infection [6,50]. These include an unspliced RNA transcript (isoform A), a single spliced RNA isoform retaining the intron 1 (isoform B), a single spliced isoform retaining the intron 2 (isoform C), a double-spliced isoform (isoform D), and a shortest isoform derived from RNA splicing of the intron 1 donor site to the intron 2 acceptor site (isoform E) (**Fig 10B**).

Our RNA-seq analysis revealed a significant shift in ORF70/K3 RNA splicing between the WT and 57KO genomes during KSHV lytic infection. In BCBL-1 cells with the WT genome, ORF70/K3 RNA predominantly uses the intron 2 splicing to avoid disruption of the ORF70 ORF, with splicing efficiency of the intron 1 being ~5-fold less than that of the intron 2 (691 intron 1-spliced reads vs 3393 intron 2-spliced reads). On the contrary, in the cells with the 57KO genome, we observed almost equal splicing efficiency for both intron 1 and 2 (1839 intron 1-spliced reads vs 2048 intron 2-spliced reads), indicating a 2.66-fold increase of the intron 1 splicing efficiency but a 1.67-fold decrease of the intron 2 splicing efficiency in the 57KO cells. However, the splicing from the intron 1 5′ ss to the intron 2 3′ ss (SJ-19) appeared only a minimal change between the WT genome (26 reads) and 57KO genome (38 reads) (**Table 1**). Subsequently, we compared the abundance of individual isoforms by Northern Blot on total RNA extracted from the WT and 57KO genome in BCBL-1 cells with lytic replication to confirm these data. First, we use an antisense oligo probe derived from exon 2 (oVM441) to detect all predicted isoforms except isoform E. As shown in **Fig 10C**, three major RNA isoforms were detected from the WT genome, corresponding to the predicted RNA isoforms A (2.5 kb), B (2 kb), C (2 kb), and D (1.5 kb), with the 2 kb transcripts being the most prevalent. Two minor RNA species, between ~3–4 kb, are presumably expressed from the upstream promoters as reported [6,50]. However, in the cells with the 57KO genome, we detected only a single 1.5 kb RNA band corresponding to the double-spliced isoform D (**Fig 10C**). Since this probe could not differentiate the RNA isoform B from the isoform C due to their similar sizes, we reprobed the same membrane with the oligo probe from the intron 1 (oVM476). We observed two major RNA bands in the size of ~2.0 and 2.5 kb from the WT cells, but not from the 57KO cells, thus confirming their identity as the unspliced isoform A and the single spliced isoform B with retention of the intron 1 in the WT cells (**Fig 10C**). As expected, this probe cannot detect the double spliced isoform D missing both intron 1 and intron 2 (**Fig 10C**).

To investigate if ORF57-mediated regulation of ORF70/K3 RNA splicing is unique to BCBL-1 cells, we took advantage of inducible iSLK/Bac16 cells carrying a KSHV WT or 57KO genome generated using the same set of gRNAs for the 57KO in BCBL-1 cells [21]. Similar to BCBL-1 cells, we were able to detect the corresponding RNA isoforms A, B/C, D, and E in the cells carrying the WT genome, but only the isoform D in the cells carrying the 57KO genome (**Fig 10D**). These data reconfirmed that ORF57 regulates ORF70/K3 RNA splicing in the context of KSHV genome in iSLK/Bac16 cells.

### KSHV ORF57 binds ORF70/K3 bicistronic RNA to regulate its RNA splicing

KSHV ORF57 is a viral RNA-binding protein that binds to its target RNAs via its disordered N-terminal domain [51,52]. To determine if ORF57 binds to ORF70/K3 bicistronic RNA to regulate its splicing, we first analyzed our recently published ORF57 CLIP-seq data representing the ORF57-associated RNAs in BCBL-1 cells during the viral lytic infection [53]. We identified the ORF57-bound ORF70/K3 RNA in the ORF57 CLIP-seq. Interestingly, among ORF57 CLIP-seq reads mapped to ORF70/K3 locus, the highest number of reads-peak covers a 75-nt region in the intron 1 of ORF70/K3 RNA (**Fig 10E**), 53 nts upstream of the nt 20263 3′ ss.

To further confirm ORF57 direct binding to ORF70/K3 RNA, we performed RNA-immunoprecipitation (RIP) using an anti-ORF57 antibody to immunoprecipitate the UV cross-linked RNA-protein complexes in the cell lysate prepared from BCBL-1 cells with KSHV lytic infection reactivated by valproic acid (VA) treatment for 24 h. The ORF70/K3 RNA in the RIP complex was RT-PCR amplified using ORF70-specific primers oVM441 and oVM481 spanning over the peak region identified by CLIP-seq. As shown in **Fig 10F**, we demonstrated that the anti-ORF57 antibody, but not a non-specific IgG control, efficiently pulled down the ORF70/K3 RNA.

Given a profound role of ORF57 in ORF70/K3 splicing, we next tested if restoration of ORF57 expression will rescue the isoform B expression in the 57KO cells. We transfected the iSLK/Bac16 57KO cells with an increased amount of the ORF57-expression vector, followed by the induction of the viral lytic infection by combined butyrate and doxycycline treatment. We used the corresponding amount of empty vector (pcDNA 3.0) as a negative control. The total RNA collected 24 h after induction was analyzed in Northern blot analysis. As expected in **Fig 10G**, the WT cells expressed mainly the B/C isoforms, while the D isoform was the most abundant in the 57KO cells. Most importantly, we found that ectopic ORF57 expression led to partially rescue the isoforms B/C expression from the 57KO genome in a dose-dependent manner. These data provide the strong evidence of ORF57’s role in regulation of ORF70/K3 RNA splicing.

K3 was proposed to be a viral immediate early gene [6, 50] and ORF57 is a viral early gene whose expression gradually increases during KSHV lytic cycle [54]. To see how ORF70/K3 RNA splicing correlates with ORF57 expression, we performed a time-course study in the WT BCBL-1 cells after induction of lytic KSHV infection by valproic acid and collected total cell RNA at 12, 24, and 36 h after VA induction to monitor the dynamic production of ORF70/K3 RNA isoforms by Northern blot analysis using an oVM441 probe described in **Fig 10B**. As shown in **Fig 10H**, all three RNA bands representing isoforms A, B/C, and D were detectable at 12, 24, and 36 h; however, their ratio displaced a substantial change between 12 and 24 h, from an approximate proportion 1.2 to 1 of isoforms D to B/C at 12 h to only 0.4 to 1 at 24 h, in favor of isoform B/C production in BCBL-1 cells with lytic KSHV infection. Consistently, this ORF57-induced isoform switch of ORF70/K3 RNAs was reproducible in iSLK/Bac16 cells with KSHV lytic infection in the other time course studies.

### KSHV ORF57-mediated ORF70/K3 RNA splicing regulates the production of thymidylate synthase ORF70 and viral E3-ubiquitin ligase K3

**Fig 11:**
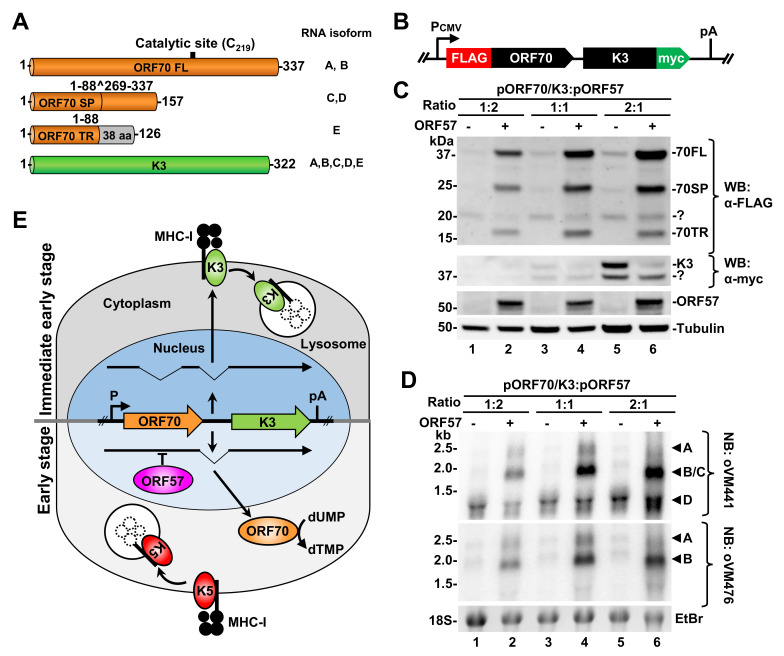
Ectopic ORF57 inhibits alternative splicing of bicistronic ORF70/K3 RNAs to regulate the expression of viral thymidylate synthase ORF70 and viral E3-ubiquitin ligase K3 in HEK293T cells. (A) A number of the amino acid residues of full length (FL), spliced (SP), truncated (TR) ORF70 and K3 proteins expressed from the corresponding RNA splicing isoforms are shown on the right (see details in **Fig 10B**). (B) Diagram of an ORF70/K3 minigene with a FLAG-tag on the ORF70 N-terminus and a c-myc-tag on the K3 C-terminus. The minigene under control by a CMV IE promoter contains the viral genomic DNA fragment covering the K3 and ORF70 coding regions and their intergenic region to ensure RNA splicing of this bicistronic RNA transcript. (C and D) Western (C, WB) and Northern blot (D, NB) analyses of HEK293T cells transfected with various ratios of the ORF70/K3 minigene over the ORF57 expression (+) or empty (-) vectors. (C) Expression of ORF70 protein was detected by WB using an anti-FLAG and K3 protein using an anti-c-myc antibody. ?, unknown protein band. (D) The changes in ORF70/K3 RNA splicing from the minigene vector in HEK293T cells in the absence (-) or presence (+) of ectopic ORF57 expression were monitored by Northern blot analysis on total cell RNA with two separate oligoprobes shown in **Fig 10B**. The 18S rRNA level detected by ethidium bromide (EtBr) staining was used as a loading control. (E) A diagram depicting the role of ORF57 in the regulation of ORF70/K3 RNA alternative splicing to mediate a switch from K3 to ORF70 protein expression during KSHV lytic replication.

Given the bicistronic nature of ORF70/K3 RNA, we next investigated how its RNA splicing regulated by ORF57 affects the expression of individual proteins. As mentioned above, ORF57-sensitive intron 1 lies entirely within the ORF70 coding region. Thus, only the RNA isoforms A and B retaining intron 1 could express a 337-aa full-length ORF70 protein (ORF70FL, **Fig 11A**). The RNA isoforms C and D lacking intron 1 would express a truncated or spliced ORF70 (ORF70SP) in size of 157 aa residues. ORF70SP lacks the central domain between aa 88 to 269, which bears a catalytic site of thymidylate synthase [55]. The isoform E potentially encodes a further 126 aa-long truncated ORF70 (ORF70TR), consisting of only the N-terminal 88 aa residues encoded by the first exon fused with additional 38 aa residues expressed from different frame (**Fig 11A**). Interestingly, the K3 coding region for viral E3-ubiquitin ligase remains intact in all spliced RNA isoforms.

Due to the lack of available antibodies against ORF70 and K3, we cannot directly measure the effect of observed regulation of ORF70/K3 alternative RNA splicing by ORF57 on the expression of ORF70 and K3 proteins. Therefore, we constructed a minigene reporter by cloning the ORF70/K3 locus into a mammalian expression vector to monitor their expression by introducing two different epitope tags: FLAG-tag on the ORF70 N-terminus and c-myc on the K3 C-terminus (**Fig 11B**). The ORF70/K3 minigene was then transfected into HEK293T cells in various ratios with a control empty or an ORF57-expressing vector. Twenty-four after transfection, the cells were harvested and blotted with an anti-FLAG antibody to detect ORF70 and an anti-myc antibody for K3 protein detection. As shown in **Fig 11C**, the minigene in the absence of ORF57 mainly expressed K3 protein and also a small amount of ORF70 protein (lanes 3 and 5). On the contrary, ORF57 co-expression switched from K3 expression to all three isoforms of ORF70 protein, with ORF70FL being the most abundant (**Fig 11C**). The efficiency of the switch was dependent on the ORF57 level and was most prominent at the 2:1 ratio of the minigene plasmid versus the ORF57-expression vector (compare lanes 2, 4, and 6).

We next performed the Northern blot analysis of the corresponding total RNA samples using the same antisense oligoprobes described in **Fig 10B**. As shown in **Fig 11D**, in the absence of ORF57, we found a double-spliced isoform D as a major RNA isoform expressed from the minigene (lanes 1, 3, and 5), which could be confirmed by both exon 2 (oVM441) and intron 1 (oVM476) probes. In contrast, co-expression of ORF57 was found not only to stabilize all isoform RNAs expressed from the minigenes as reported [22, 26], but also to substantially increase the single spliced isoform RNA B and to slightly increase the unspliced pre-mRNA (isoform A) (lanes 2, 4 and 6). Together, we demonstrated that ORF57-mediated splicing of the minigene-derived ORF70/K3 RNA is responsible for controlling the expression levels of viral thymidylate synthase ORF70 and viral E3-ubiquitin ligase K3, which is independent of other viral factors.

## Discussion

In this study using RNA-seq analysis combined with bioinformatics and multiple experimental approaches, we performed a comprehensive investigation of viral RNA splicing events associated with KSHV gene expression in BCBL-1 cells carrying a viral WT or a 57KO genome. In addition to detection of all RNA splicing events in the reported gene transcripts [8,9,14,30], we identified hundreds of splicing events in the expression of both WT and 57KO genomes in BCBL-1 cells with KSHV lytic infection and experimentally confirmed most of the newly annotated RNA splicing events. By comparing viral RNA splicing events in the expression of the WT over 57KO genome in BCBL-1 cells, we uncovered the roles of ORF57 in regulation of KSHV RNA splicing and identified many viral genes as a new target for ORF57-mediated RNA splicing regulation to control KSHV protein expression during lytic infection.

The patient-derived primary effusion (PEL) cell lines represent naturally infected, transformed B-cells used as a model for studying KSHV biology, with BCBL-1 cells being one of the most widely used due to the absence of EBV coinfection observed in many other PEL cells [20, 56]. However, the high number of viral genome copies (≥ 100 copies per cell) makes the cell line limited usage in KSHV genetic studies. We recently developed a modified CRISPR/Cas9 technology allowing the rapid generation of viral ORF57 gene KO from all viral genome copies in selected single-cell clones [21]. These single-cell clones with the 57KO genome allowed us for the first time to study ORF57’s role in viral genome expression during native KSHV infection, thus providing an advantage over the studies conducted in BAC-based recombinant KSHV genome-transfected cell lines [24,57]. Although ORF57 has been demonstrated to stabilize PAN RNA, we found that the selected 57KO clone #6 cells appear compensating the expression of genes highly sensitive to post-transcriptional regulation by ORF57, such as PAN of which expression was found just 7-fold lower by RT-qPCR than the PAN level in the WT cells during viral lytic infection. The mechanism of such compensation in viral gene expression in the 57KO clone #6 cells remains to be investigated.

RNA splicing represents an important step in the processing of both eukaryotic and viral pre-mRNAs and is prevalent in the production of numerous KSHV mRNA transcripts [9,10,14,30]. However, the scale, regulation, and biological roles of KSHV RNA splicing remain poorly understood. We identified hundreds of KSHV RNA splice junctions, mainly in the expression of viral lytic genes and found 57 highly confident splicing events detected from the WT genome and an additional 20 from the 57KO genome. The newly identified RNA splicing events were found in both split and well-known intronless transcripts. Among ~30% KSHV split genes undergoing RNA splicing in the virus genome, the majority are KSHV-specific genes (K1-K15) encoding host mimics to tackle host responses during virus infection. These genes presumably originated from the host genome and the high splicing frequency detected in their RNA transcripts may represent an evolutionary memory of their cellular origin. On the contrary, the transcripts from other viral genes being highly conserved in other herpesviruses mostly avoid RNA splicing.

Although the newly annotated RNA splicing events in the coding regions of ORF2, K4.2, ORF45-47, and K12 may lead to the expression of potentially new protein isoforms, other mapped splicing events occur in the untranslated regions (UTR), such as the 5′ and 3′ UTRs or in the intergenic regions, thus providing an additional layer of regulation of viral gene expression. For example, we observed extensive alternative RNA splicing from the 5′ UTR of viral K5 and K6 transcripts that harbor several upstream ORFs (uORF) or small ORFs (sORF) [9]. The uORFs and sORFs have been shown to regulate the expression of both host and viral RNAs by stimulating or suppressing the translation of downstream ORFs [58–60]. How the RNA splicing in the 5′ UTRs of viral transcripts contributes to their expression remains to be determined. Additional alternative RNA splicing from the K6 5′ UTR to K5 RNA allows K5 expression from a K6 promoter and thus prevents the K6 promoter-derived transcripts from polyadenylation at a pA site mapped downstream of the K6 ORF [8].

KSHV has a common strategy to express a cluster of genes using a gene-specific promoter, but all RNA transcripts transcribed from these different promoters are polyadenylated at a single pA site [8]. The use of this strategy is well documented in the expression of RTA, K8, and K8.1, or ORF48, ORF47, ORF46, and ORF45 genes [61,62]. In this case, the mapped RNA splicing occurs within transcripts’ 3′ UTRs. Here RNA splicing may be required to remove the suppressive and toxic sequences, such as the binding site for host miRNAs or RNA-binding proteins, to promote expression of these polycistronic transcripts [63]. It may also prevent the formation of extended double-stranded RNA structures in a long 3′ UTR recognizable by host RNA sensors which could induce innate immune responses. Interestingly, we recently found that KSHV developed two important mechanisms to counteract these processes by ORF57-mediated inhibition of P-bodies and stress granules [64,65].

We have identified a group of splice junction reads spanning large parts of the viral genome (≥10 kb) across multiple transcriptional units. This long-range RNA splicing happens in both plus-strand RNA transcripts and minus-strand RNA transcripts, predominantly in the same transcription direction but also in the opposite direction of RNA transcripts. Some of these splicing events are consistently detected in different KSHV-infected cells, including JSC-1 and iSLK/Bac16 cells, further supporting their authenticity. The biological relevance of this long-distance RNA splicing remains to be investigated. The coding potential analysis identified several short novel ORFs crossing the splice junction from these transcripts derived by long-distance RNA splicing. Despite their low abundance, the biological relevance of these novel ORF-encoded small proteins also needs to be studied.

Although the presented study focuses on profiling KSHV genome-wide RNA splicing of all viral RNA transcripts from RNA 5′ to 3′ direction (canonical splicing), recent reports suggested the presence of detectable RNA back-splicing from RNA 3′ to 5′ direction in KSHV transcripts leading to expression of viral circular RNAs [35–37]. Using a circRNA prediction pipeline CIRI2 [38, 39] to analyze our RNA-seq library reads, we predicted only 8 unique KSHV circRNAs in BCBL-1 cells with lytic replication, six from the WT genome and two from the 57KO genome, mainly from the antisense PAN (or K7.3) [66]. Despite that both sense and antisense transcripts from the PAN locus were not previously considered to be spliced, we observed several rare splicing events in PAN and K7.3 transcripts more often in the 57KO cells but did not find any of these rare splicing events being used for production of the predicted PAN circRNAs. Experimentally, we could not detect these PAN- or K7.3-derived circRNAs in BCBL-1 cells with lytic KSHV infection by RT-PCR using inverse primer pairs as reported [35]. The prediction of K7.3 circRNAs was even more surprising since, in contrast to the highly expressed PAN transcript, the expression of this antisense transcript was only minimal in the cells with lytic KSHV infection. The inconsistency among different studies and lack of functional splice sites further suggest that all PAN circRNAs predicted in the recent reports might be the artifacts of RNA-seq library preparation from the erroneously ligated chimeric reads and/or the software used for circRNA prediction [67].

On the contrary, the back-splicing reads detected from vIRF4 (WT-circRNA-6) locus were real and more comparable to the number of linear RNA splice junction reads (ratio 1:5 in the WT cells) and the overall transcription level. Its 5′ end overlaps with the 3′ acceptor site of vIRF4 intron 1 (SJ-43), being spliced to a new 5′ donor site downstream of intron 2. We confirmed the production of vIRF4 circRNA in the WT B4 cells during KSHV lytic infection by RT-PCR using an inverse primer pair next to the back-splice sites. Interestingly, the production of this circRNA was related to ORF57 expression. We found that the reduced expression of vIRF4 circRNA was correlated with the reduction of linear vIRF4 RNA splicing in the 57KO #6 cells. However, the active role (s) of ORF57 in the biogenesis of viral vIRF4 circRNA remains unknown.

The regulation of KSHV splicing is poorly understood. Viral transcripts are spliced by cellular RNA splicing machinery and thus subject to similar regulatory mechanisms as host RNAs, including splice site selections, RNA *cis*-elements in interaction with host splicing factors, etc. [33]. We previously identified viral RNA-binding ORF57 protein as a viral splicing factor promoting the alternative RNA splicing of several early lytic genes, including ORF50 and K8 [17]. ORF57 regulation of K8 RNA splicing was found to be independent of other viral proteins but required ORF57 binding to host splicing factor SRSF3 to attenuate its suppressive activity on splicing of the K8 intron 2 [18]. However, we did not see this was happening in the 57KO clone #6 cells with remaining expression of high amount of PAN RNA and decreased level of host SRSF3. We subsequently found the increased splicing of K8 intron 2 by overexpressed PAN RNA in HEK293T cells presumably by sponging SRSF3 (S3A Fig).

Two most remarkable effects of ORF57 on KSHV RNA splicing were identified for RNA processing of monocistronic K15 and bicistronic ORF70/K3 RNA transcripts. K15 gene comprises eight exons and seven introns [14] and encodes a non-structural membrane-bound protein responsible for KSHV-induced angiogenesis and productive infection [43, 47]. We found that the 57KO genome displayed more efficient K15 splicing of the first two introns, both containing a premature termination codon. Their retention, clearly observed from the WT genome in the cells, would reduce the expression of full-length K15 protein. We also observed alternative RNA splicing of K15 RNA, leading to the production of various spliced RNA isoforms as previously reported, but none of these RNA isoforms had enough splice junction reads over the threshold ≥10 reads. In the WT B4 cells, we observed K15 exon 5 skipping and alternative 3′ ss usage in K15 exon 8. In the 57KO #6 cells, we found splicing from an alternative 5′ ss in the first exon to a 3′ ss of intron 3. These data clearly indicate that ORF57 regulates RNA splicing and expression of the K15 gene to fine-tune its activity.

Bicistronic ORF70/K3 RNA transcript encodes viral thymidylate synthase (ORF70) and viral membrane-bound E3-ubiquitin ligase (K3), each being expressed from an individual ORF separated by a 480-nt long intergenic region. In accordance with previous reports, we detected multiple spliced isoforms of ORF70/K3 RNA generated by alternative splicing of two major introns, with a suboptimal intron 1 in the ORF70 coding region and a strong intron 2 within the intergenic region. We found that the intron 1 is alternatively spliced and ORF57 strongly inhibits its splicing. The intron 2 is a constitutive intron not susceptible to ORF57 regulation. ORF57 was found to bind the intron 1 region right upstream of its weak 3′ ss, consistent with ORF57 binding to the K8 suboptimal intron 2 [18]. The dependence of ORF70/K3 splicing on ORF57 was further supported by a time-course study showing a direct correlation in the ratio change of alternatively spliced isoforms on the level of ORF57 during the KSHV lytic infection. The KSHV 57KO genome in BCBL-1 and iSLK/Bac16 cells also exclusively expresses only a double spliced isoform D. A similar switch in spliced RNA isoforms of ORF70/K3 RNA expression was reported after treating BCBL-1 cells with cycloheximide, a protein synthesis inhibitor, to suppress the expression of early viral genes, including ORF57 [50]. However, the switch in production of individual ORF70/K3 isoforms in the reported study [50] was wrongly interpreted as a result from alternative promoter usage.

Functionally, the intron 1 retention in the presence of ORF57 is necessary for the expression of ORF70 during viral lytic infection, but the double spliced RNA isoform D is responsible for K3 protein production. Using an ORF70/K3 minigene, we confirmed that the double spliced RNA isoform D expresses K3 protein, while the intron 1 retention in the ORF70 ORF region mediated by ORF57 led to the expression of ORF70 protein. The biological significance of KSHV ORF57-mediated regulation of the expression of ORF70 and K3 is highlighted in **Fig 11E**. In the absence of ORF57, K3 expressed from the double spliced bicistronic ORF70/K3 RNA as a viral immediate-early protein functions as an E3-ubiquitin ligase which promotes KSHV immune evasion by degrading host MHC-I proteins to establish productive viral infection [68]. The rising expression of ORF57 in the early stage of virus infection blocks the intron 1 splicing to promote the production of viral thymidylate synthase (ORF70) for robust viral DNA replication by mediating dTMP synthesis [49]. As reported, the expression of structurally and functionally homologous K5 protein can compensate for the loss of K3 expression at later infection [69,70]. Nevertheless, we found that ORF57 exhibits both stimulatory and suppressive activity on viral RNA splicing and splice site selection, a similar dual effect on RNA splicing which was also observed for ORF57 homolog ICP27 from herpes simplex viruses [71,72].

In conclusion, we have identified KSHV ORF57 as a viral splicing factor in the global regulation of KSHV genome expression and RNA splicing. The ORF57-mediated splicing regulation profoundly affects the expression of affected genes as shown in the expression of viral thymidylate synthase ORF70 and viral E3-ubiquitin ligase K3 during the KSHV life cycle, thus providing further insight into KSHV biology.

## Materials and methods

### Cell cultures

KSHV-positive BCBL-1 [20] and JSC-1 [73] cells were cultivated in RPMI 1640 medium (Thermo Fisher Scientific). The iSLK cells carrying KSHV Bac16 genomes [69] and HEK293T (ATCC) cells were cultivated in a DMEM medium (Thermo Fisher Scientific). All media were supplemented with 10% fetal bovine serum (FBS, Hyclone, Cytiva) and 1 × Penicillin-Streptomycin-Glutamine (Thermo Fisher Scientific). Hygromycin B (150 μg/ml), G418 (250 μg/ml) and 1 μg/ml puromycin were added to the culture media of iSLK/Bac16 cells to maintain the KSHV genome. The ORF57 knock-out (57KO) in BCBL-1 and iSLK/Bac16 cells was generated by a modified CRISPR/Cas9 technology as described [21]. The single clones of parental cells transfected with a plasmid expressing Cas9 lacking gRNA expression were also selected in parallel and used as control wild-type (WT) cells. To reactivate KSHV lytic replication, BCBL-1 and JSC-1 cells were treated with 1 mM final concentration of sodium salt of valproic acid (VA, Sigma-Aldrich) or 3 mM sodium butyrate (Bu, Sigma-Aldrich), respectively, for 24 h. KSHV replication in iSLK/Bac16 cells was reactivated by a combination of 1 μg/ml doxycycline (DOX, Clontech) and 1 mM sodium butyrate (Bu, Sigma-Aldrich). RNA samples were collected 24 h after the reactivation.

### RNA-seq and ORF57 CLIP-seq data analysis

Total RNA from 4 replicates of BCBL-1 WT clone B4 and 57KO clone #6 cells were isolated by TriPure Reagent (Roche) 24 h after induction with VA. The genomic DNA was removed using RNeasy Mini Kit (Qiagen) on-column DNase-treatment. Total RNA-seq libraries were prepared with TruSeq Stranded Total RNA Library Kit and subjected to pair-end sequencing using HiSeq3000/4000 chemistry and 2 × 150 nts modality. Reads were trimmed with CutAdapt v.1.18 and mapped to human GRCh38 (hg38)/KSHV (GenBank Acc. No. HQ404500) chimeric genome using STAR aligner v.2.7.0f. [23]. The changes of viral gene expression between individual groups were determined by the Limma Voom package using reads mapped to annotated KSHV ORFs. The splice reads mapped to the KSHV genome were extracted to identify unique viral splice junctions. The distribution of splicing junction along the KSHV genome was visualized using Integrative Genomics Viewer (IGV, Broad Institute). The strength of viral splicing sites was calculated using the MaxEntScan::score algorithm [32]. The original sequence libraries were deposited in NCBI’s GEO repository (Acc. No. GSE194239). ORF57 CLIP-seq was detailed in our previous publication [53] and the obtained sequence libraries are publicly available in NCBI’s GEO depository (Acc. No. GSE179726). The obtained reads were mapped to chimeric human GRCh37 (hg19)/KSHV (GenBank Acc. No. U75698) reference genome using STAR aligner (version 2.7.6a) [23] and visualizes by IGV.

### RNA-immunoprecipitation (RIP)

To isolate ORF57 bound RNAs, we performed a RIP assay as previously described [53]. Briefly, BCBL-1 cells treated with 1 mM valproic acid (VA) for 24 h were exposed to 480 mJ/cm^2^ UV light of wavelength 254 nm. The cross-linked cells were lysed in the radioimmunoprecipitation assay buffer (RIPA, Boston Bioproducts) and the obtained cell extract was used for immunoprecipitation with protein A-agarose beads (Millipore-Sigma) coated with the rabbit anti-ORF57 antibody [51] or a control non-specific rabbit IgG (Thermo Fisher Scientific). The pulled-down RNA was released by proteinase K, extracted by phenol:chloroform, and precipitated. The extracted RNA was used for RT-PCR detections.

### RT-PCR and RT-qPCR

Before RT, the total RNA isolated by TriPure Reagent (Roche) was treated with Turbo DNase (Thermo Fisher Scientific). The specific products were amplified using two-step RT-PCR using random hexamers and primers listed in the **S6 Table**. The reactions without reverse transcriptase were used as the negative controls. The RT-PCR products were separated in agarose gel, purified, and the splicing junctions were verified by Sanger sequencing. For RT-qPCR, the DNase-treated RNAs were converted to cDNAs by SuperScript First-Strand Synthesis System (Thermo Fisher Scientific) using random hexamers as recommended. The resulting cDNAs were used as templates in qPCR reactions containing TaqMan Universal PCR Master Mix (Thermo Fisher Scientific) and custom PrimeTime qPCR assay (IDT) for KSHV PAN and ORF59 RNAs [25,26]. The cellular GAPDH level detected by the TaqMan assay (Hs02758991_g1, Thermo Fisher Scientific) was used for the normalization. All amplifications were carried out on StepOne Real-Time PCR System (Thermo Fisher Scientific). The changes in expression level between individual samples were determined by the ΔΔC_t_ method.

### Northern blot

Total RNAs isolated by TriPure reagent were separated along with Millennium RNA marker (Thermo Fisher Scientific) in formaldehyde-containing 1% agarose gel in 1 × MOPS buffer. After transfer, the membranes were hybridized overnight with antisense oligo probes (**S6 Table**) end-labeled with T4-PNK (New England Biolabs) using ^32^P-γ-ATP (Perkin Elmer). Washed membranes were exposed to a phosphor imaging screen and scanned by GE Typhoon TRIO Imager (GE Healthcare). Obtained images were processed by Image Lab software (BioRad).

### Plasmids and reporter plasmid construction

The construction of K8 splicing reporter pST3, containing KSHV K8β cDNA, was described in [29]. The plasmid pJM1 was generated by cloning KSHV PAN RNA into pcDNA3.0 (Invitrogen) [26]. KSHV ORF57 expression vector pORF57 [40] was used to rescue ORF57 expression in the iSLK/Bac16 57KO cells. The empty vector pcDNA3.0 (Invitrogen) used a negative control. To generate a minigene system for monitoring ORF70/K3 RNA splicing and protein expression (pVM140), the ORF70/K3 locus was amplified with oVM454 and oVM455 primers (see **S6 Table** for details) on BCBL-1 total DNA allowing its in-frame cloning into pCMV-FLAG-myc-22 (Sigma-Aldrich) using *Kpn*I and *Not*I restriction sites. All plasmid transfections were performed by LipoD293 In Vitro DNA transfection reagent (SignaGen Laboratories) as recommended.

### Western blot

Total proteins, prepared by cells direct lysis in SDS protein sample buffer, were separated in 4–12% Bis-TRIS NuPAGE gels (Thermo Fisher Scientific) in 1 × MES buffer and transferred onto a nitrocellulose membrane. The in-house produced anti-ORF57 polyclonal antibody was used to ORF57 expression [51]. The anti-ORF50 antibody was generously provided by Dr. Yoshihiro Izumiya (University of California, Davis). The anti-Cas9 mouse monoclonal antibody was purchased from Cell Signaling (cat. no. 14697). The anti-FLAG polyclonal antibody (cat. no. F7425, Sigma-Aldrich) was used for FLAG-ORF70 and anti-c-myc clone 9E10 antibody (a generous gift from Dr. Xuefeng Liu, Georgetown University) for K3-myc fusion detection in the reporter assay. Mouse monoclonal anti-K8 antibody were obtain from ProMab Biotechnologies (cat. no. 20015). In-house generated culture media from the mouse hybridoma clone (7B4) culture expressing anti-SRSF3 antibody was used for SRSF3 detection. The cellular β-tubulin detected by mouse monoclonal antibody (cat. no. T5201, Sigma-Aldrich) was used as a loading control. The secondary horseradish peroxidase-labeled secondary antibodies (Sigma-Aldrich) were used for signal development using ProSignal Pico ECL Reagent (GeneSee Scientific) captured by Chemidoc Touch Imager (BioRad).

### Prediction and detection of circRNAs

KSHV circRNAs were predicted by CIRI2 pipeline (https://sourceforge.net/projects/ciri/) [38,39] with default setting using RNA-seq reads from BCBL-1 WT B4 and 57KO #6 cells. To experimentally confirm viral circRNAs expression, two μg of total RNA were treated with 5 U of RNase R (Lucigen) for 40 min at 37°C. The reactions containing the corresponding amount of RNA without RNase R treatment served as a negative control. The obtained RNAs were reverse transcribed with SuperScript IV (Thermo Fisher Scientific) at 50°C for 1 h in the presence of random hexamers, followed by detection of circular RNA as described [74]. For circRNA detection, 500 ng of cDNA were amplified by 40 PCR cycles using an inverse primer pair and SuperFi II DNA polymerase (Thermo Fisher Scientific). The linear RNAs were amplified using 100 ng of cDNA for 25 cycles of PCR amplification using a corresponding primer pair. All primers are listed in the **S6 Table**.

## Supporting information

S1 Fig Selective validation of the newly identified splice junctions in BCBL-1 cells with a WT KSHV genome. Total RNA from BCBL-1 cells without (VA-) or with (VA+) virus lytic replication was used as an RT-PCR template. SJ-43+42 means a RT-PCR product from the double RNA splicing first from the splice junction SJ-43 and then SJ-42 (see **Fig 4**). Numbers in parenthesis are the RNA splice junction reads detected from the cells with KSHV latent (-VA)/lytic (+VA) infection. See primer’s information in **Figs 3 and 4 and S6 Table**.
(TIF)

S2 Fig RT-PCR validation of selected RNA splicing events from the 57KO genome in BCBL-1 cells. Total RNA from the WT and 57KO KSHV genome containing BCBL-1 cells treated with valproic acid (VA) for 24 h to induce KSHV lytic replication was used for RT-PCR to detect viral RNA splicing events from the 57KO (A) and WT KSHV genome (B) or splicing events with altered splicing efficiency from the WT to 57KO KSHV genomes (C). SJ-61+6 means a RT-PCR product from the double RNA splicing first from the splice junction SJ-61 and then SJ-6, and SJ-17+21 first from SJ-17 and then SJ-21 (see **Figs 3 and 4**). Numbers in parenthesis are the viral RNA splice junction reads detected from the WT/57KO cells during KSHV lytic (+VA) infection. See primer’s information in **Figs 3 and 4 and S6 Table**.
(TIF)

S3 Fig KSHV PAN RNA in overexpression promotes K8 splicing in the absence of ORF57. (A) HEK293T cells were co-transfected with 300 ng of K8β cDNA plasmid (pST3) [29] with an increased amount of PAN RNA expression vector (pJM1) [26]. The same amount of an empty pcDNA3.0 vector (-) was used as a control plasmid in the cells without PAN expression. Twenty-four hours after transfection the cells were harvested and K8β splicing was monitored by Western blotting using an anti-K8 antibody. Host β-tubulin was used as a loading control. (B) Expression of SRSF3 protein in BCBL-1 cells with the WT or 57KO genome during latent (VA -) or lytic (VA +) infection was blotted with an anti-SRSF3 (7B4) monoclonal antibody. The Ponceau staining was used to determine the sample loading based on total protein amount.
(TIF)

S4 Fig Viral long-range RNA splicing creates possible novel ORFs for KSHV coding potentials. (A) The conservation of long-range KSHV splicing events in JSC-1 and iSLK/Bac16 cells. Total RNA from JSC-1 cells treated with the sodium butyrate (Bu) for 24 h or from iSLK/Bac16 cells with the KSHV WT or 57KO genome treated with sodium butyrate/doxycycline (Bu/Dox) for 48 h was used in RT-PCR with indicated primers. The reaction without reverse transcriptase (RT-) was used as a negative control. *, non-specific RT-PCR products. (B) Novel transcripts generated by long-range RNA splicing identified in KSHV lytic infection. The annotated KSHV ORFs are shown in orange and blue. The novel ORFs (green arrows) spanning the long-range splice junctions (^) shown in **Fig 7B** were predicted using an ORF prediction program (ORFfinder, NCBI) with an AUG initiation codon ORF encoding at least 50 aa or more from all three frames. The nucleotide positions and amino acid sequences of the newly identified ORFs are shown below.
(TIF)

S5 Fig Lack of confident RNA splice sites in the PAN region to support predicted PAN-derived circRNAs. (A) Diagrams of predicted circRNAs from the sense (+) or antisense (-) RNA transcripts expressed from the KSHV genome and their relations to the corresponding splice sites (ss). (B) The KSHV K7/PAN RNA locus in Bac36/BCBL-1 reference genome with the nt position and orientation of each circRNA predicted in this study (WT-circRNA-1-5) and other three reports [35–37]. The nucleotide position of each RNA splice site contributing to predicted circRNA biogenesis is shown together with the flanking dinucleotides and its splice site strength score (in parentheses) based on MAX:ENT model [32]. ND-not determined.
(TIF)

S1 Table Differential expression of KSHV genes in BCBL-1 single-cell clones carrying a WT B4 or 57KO #6 genome during latent and lytic infection determined by Limma Voom package based on the number of mapped RNA-seq reads in each group (A, B, or C) as described in Fig 1A. (XLSX)

S2 Table The summary table of all viral splice junctions detected in individual groups (A, B, or C) described in Fig 1A. (XLSX)

S3 Table The predicted KSHV circRNAs in individual RNA-seq samples described in Fig 1A from the WT and 57KO BCBL-1 cells with lytic KSHV infection by the CIRI2 pipeline [38, 39]. (XLSX)

S4 Table Strength score analysis of the splice sites for linear RNA splice junctions with at least two splice junction reads mapped to the PAN RNA region from either plus- or minus-strand of KSHV genome. The splice site strength was determined by MAX:ENT algorithm [32].
(XLSX)

S5 Table Strength score analysis of the splice sites flanking the predicted circRNAs from PAN and K7.3 RNAs with at least 1000 back-splice junction reads from [35]. The splice sites strength was determined by MAX:ENT algorithm [32].
(XLSX)

S6 Table The sequences and positions of oligos used in this study. (XLSX)
