# Supplementary figures and images for: Genome-wide regulation of KSHV RNA splicing by viral RNA-binding protein ORF57
Source: PLoS Pathog. 2022 Jul 14;18(7):e1010311. doi: 10.1371/journal.ppat.1010311 (PMC9321434; doi:10.1371/journal.ppat.1010311)

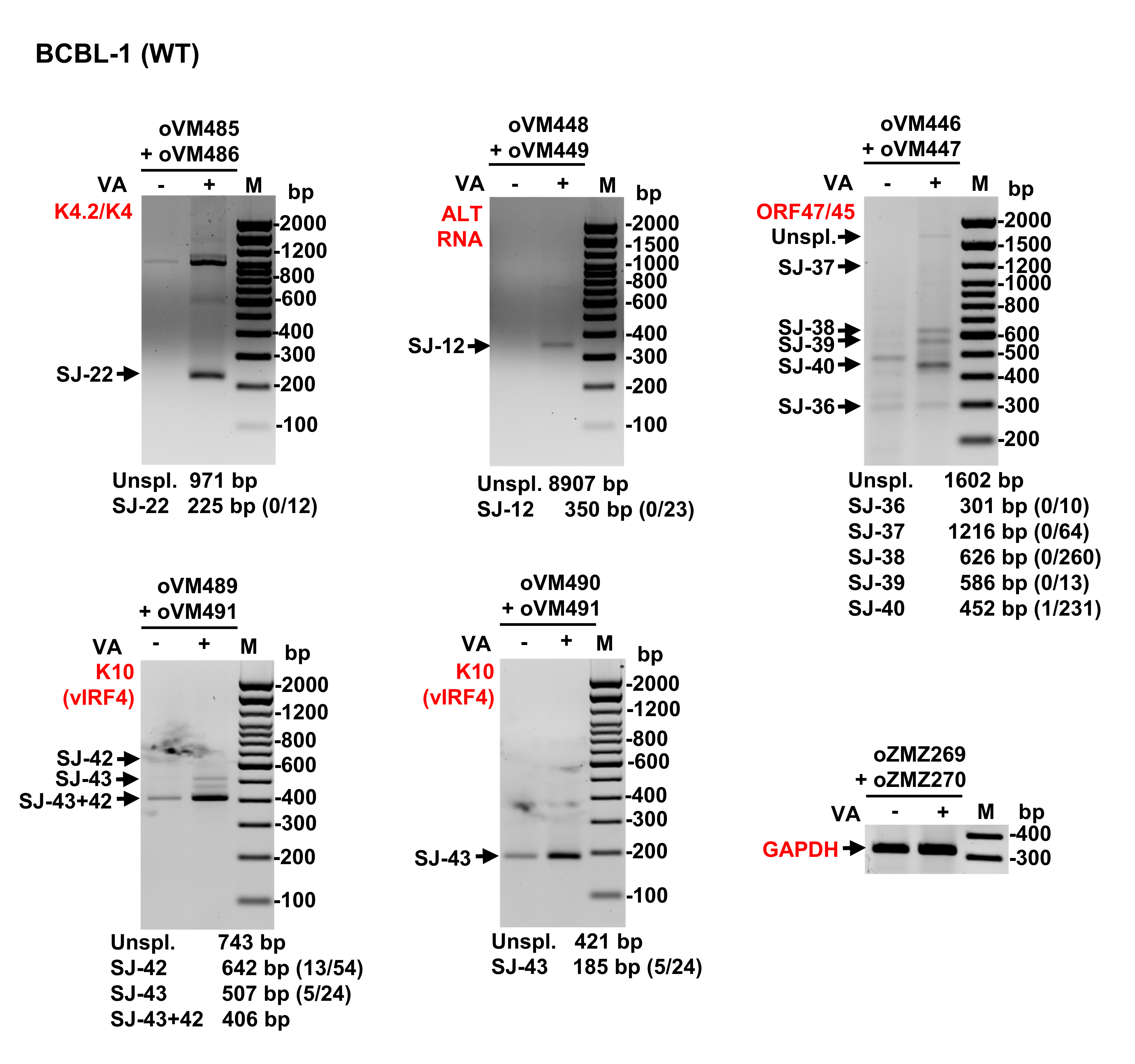

Supplement: S1 Fig — Total RNA from BCBL-1 cells without (VA-) or with (VA+) virus lytic replication was used as an RT-PCR template. SJ-43+42 means a RT-PCR product from the double RNA splicing first from the splice junction SJ-43 and then SJ-42 (see Fig 4). Numbers in parenthesis are the RNA splice junction reads detected from the cells with KSHV latent (-VA)/lytic (+VA) infection. See primer’s information in Figs 3 and 4 and S6 Table. (TIF) [file ppat.1010311.s001.tif]

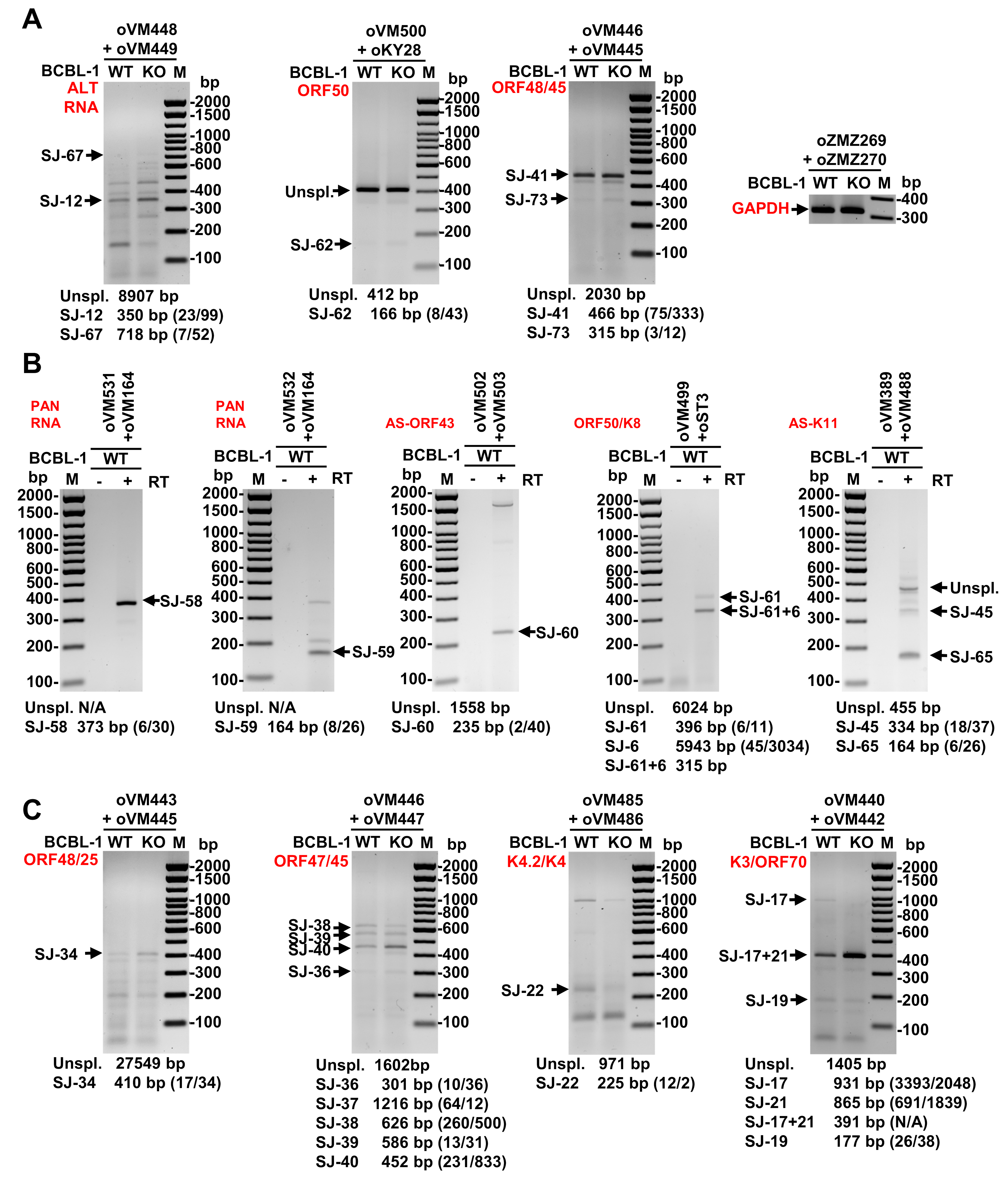

Supplement: S2 Fig — Total RNA from the WT and 57KO KSHV genome containing BCBL-1 cells treated with valproic acid (VA) for 24 h to induce KSHV lytic replication was used for RT-PCR to detect viral RNA splicing events from the 57KO (A) and WT KSHV genome (B) or splicing events with altered splicing efficiency from the WT to 57KO KSHV genomes (C). SJ-61+6 means a RT-PCR product from the double RNA splicing first from the splice junction SJ-61 and then SJ-6, and SJ-17+21 first from SJ-17 and then SJ-21 (see Figs 3 and 4). Numbers in parenthesis are the viral RNA splice junction reads detected from the WT/57KO cells during KSHV lytic (+VA) infection. See primer’s information in Figs 3 and 4 and S6 Table. (TIF) [file ppat.1010311.s002.tif]

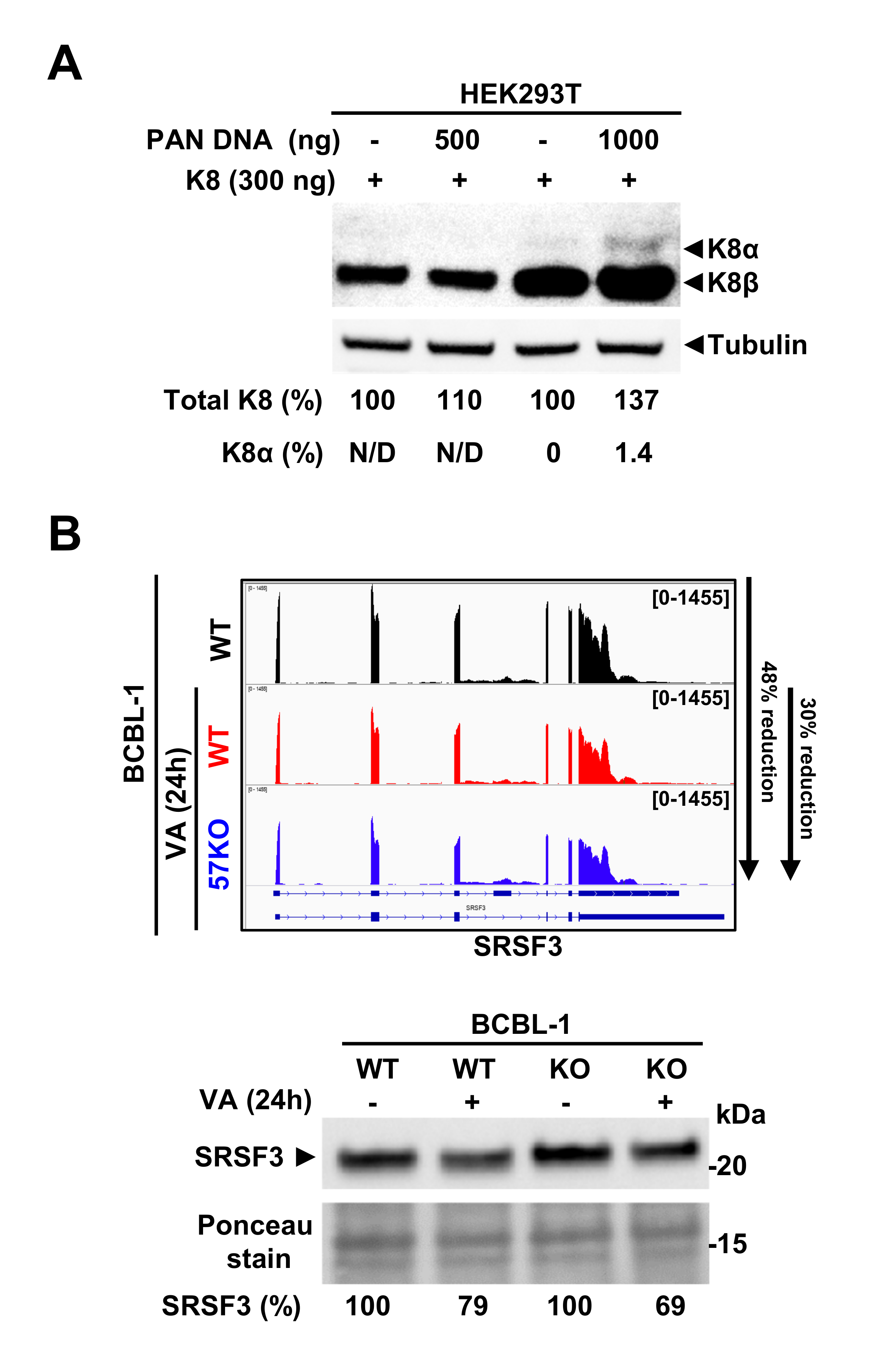

Supplement: S3 Fig — (A) HEK293T cells were co-transfected with 300 ng of K8β cDNA plasmid (pST3) [29] with an increased amount of PAN RNA expression vector (pJM1) [26]. The same amount of an empty pcDNA3.0 vector (-) was used as a control plasmid in the cells without PAN expression. Twenty-four hours after transfection the cells were harvested and K8β splicing was monitored by Western blotting using an anti-K8 antibody. Host β-tubulin was used as a loading control. (B) Expression of SRSF3 protein in BCBL-1 cells with the WT or 57KO genome during latent (VA -) or lytic (VA +) infection was blotted with an anti-SRSF3 (7B4) monoclonal antibody. The Ponceau staining was used to determine the sample loading based on total protein amount. (TIF) [file ppat.1010311.s003.tif]

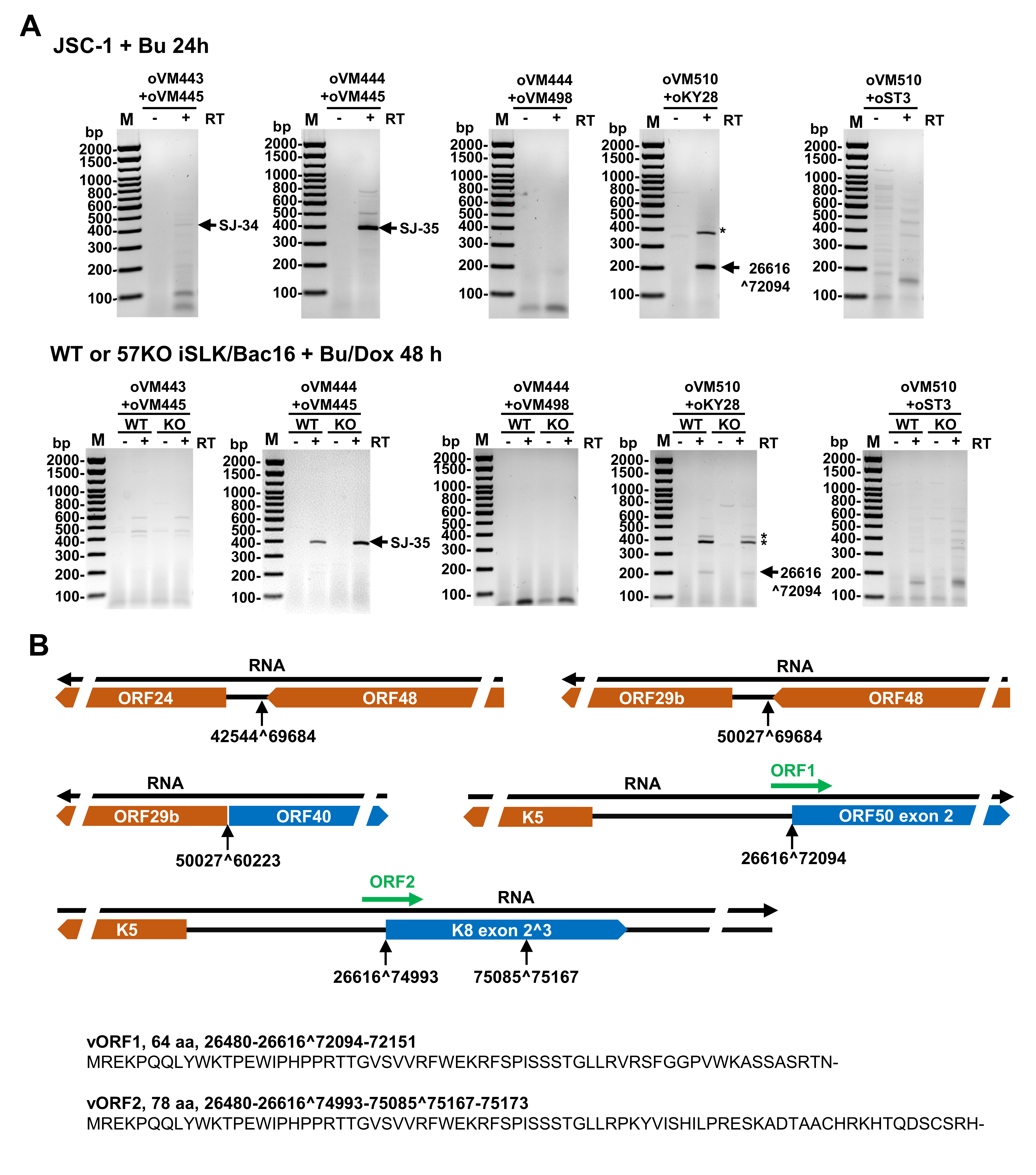

Supplement: S4 Fig — (A) The conservation of long-range KSHV splicing events in JSC-1 and iSLK/Bac16 cells. Total RNA from JSC-1 cells treated with the sodium butyrate (Bu) for 24 h or from iSLK/Bac16 cells with the KSHV WT or 57KO genome treated with sodium butyrate/doxycycline (Bu/Dox) for 48 h was used in RT-PCR with indicated primers. The reaction without reverse transcriptase (RT-) was used as a negative control. *, non-specific RT-PCR products. (B) Novel transcripts generated by long-range RNA splicing identified in KSHV lytic infection. The annotated KSHV ORFs are shown in orange and blue. The novel ORFs (green arrows) spanning the long-range splice junctions (^) shown in Fig 7B were predicted using an ORF prediction program (ORFfinder, NCBI) with an AUG initiation codon ORF encoding at least 50 aa or more from all three frames. The nucleotide positions and amino acid sequences of the newly identified ORFs are shown below. (TIF) [file ppat.1010311.s004.tif]

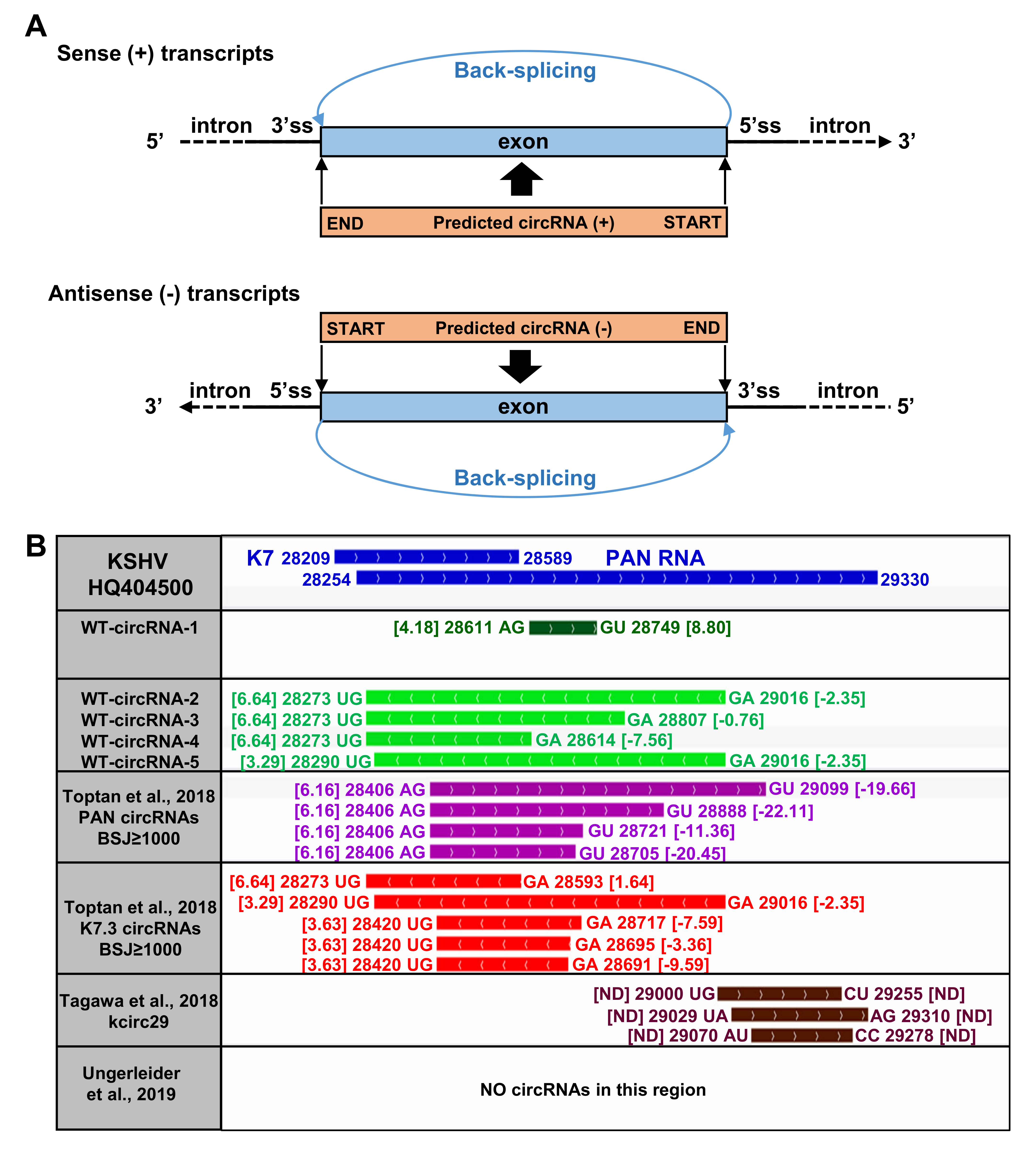

Supplement: S5 Fig — (A) Diagrams of predicted circRNAs from the sense (+) or antisense (-) RNA transcripts expressed from the KSHV genome and their relations to the corresponding splice sites (ss). (B) The KSHV K7/PAN RNA locus in Bac36/BCBL-1 reference genome with the nt position and orientation of each circRNA predicted in this study (WT-circRNA-1-5) and other three reports [35–37]. The nucleotide position of each RNA splice site contributing to predicted circRNA biogenesis is shown together with the flanking dinucleotides and its splice site strength score (in parentheses) based on MAX:ENT model [32]. ND-not determined. (TIF) [file ppat.1010311.s005.tif]
